# Structural and functional analysis of gum arabic l-rhamnose-α-1,4-d-glucuronate lyase establishes a novel polysaccharide lyase family

**DOI:** 10.1016/j.jbc.2021.101001

**Published:** 2021-07-23

**Authors:** Tatsuya Kondo, Miyu Kichijo, Akiho Maruta, Makoto Nakaya, Shigeo Takenaka, Takatoshi Arakawa, Shinya Fushinobu, Tatsuji Sakamoto

**Affiliations:** 1 Graduate School of Life and Environmental Sciences, Osaka Prefecture University, Osaka, Japan; 2 Center for Research and Development of Bioresources, Organization for Research Promotion, Osaka Prefecture University, Osaka, Japan; 3 Department of Nutrition, Otemae College of Nutrition and Confectionery, Osaka, Japan; 4 Graduate School of Comprehensive Rehabilitation, Osaka Prefecture University, Osaka, Japan; 5 Department of Biotechnology, The University of Tokyo, Tokyo, Japan; 6 Collaborative Research Institute for Innovative Microbiology, The University of Tokyo, Tokyo, Japan

**Keywords:** arabinogalactan protein, catalysis, *Fusarium oxysporum*, gum arabic, polysaccharide, polysaccharide lyase, l-rhamnose-α-1,4-d-glucuronate lyase, substrate specificity, structural biology, structure–function, AGP, arabinogalactan protein, D_2_O, heavy water, GA, gum arabic, GH, glycoside hydrolase, HPAEC-PAD, high-performance anion-exchange chromatography with a pulsed amperometric detector, IT, ion trap, LWAG, larch wood arabinogalactan, PL, polysaccharide lyase, *p*NP-α-l-Rha, *p*-nitrophenyl α-l-rhamnopyranoside, RG, rhamnogalacturonan, Rha–GlcA, α-l-rhamnose-(1→4)-d-glucuronic acid

## Abstract

Gum arabic (GA) is widely used as an emulsion stabilizer and coating in several industrial applications, such as foods and pharmaceuticals. GA contains a complex carbohydrate moiety, and the nonreducing ends of the side chains are often capped with l-rhamnose; thus, enzymes that can remove these caps are promising tools for the structural analysis of the carbohydrates comprising GA. In this study, GA-specific l-rhamnose-α-1,4-d-glucuronate lyase from the fungus *Fusarium oxysporum* 12S (FoRham1) was cloned and characterized. FoRham1 showed the highest amino acid sequence similarity with enzymes belonging to the glycoside hydrolase family 145; however, the catalytic residue on the posterior pocket of the β-propeller fold protein was not conserved. The catalytic residues of FoRham1 were instead conserved with ulvan lyases belonging to polysaccharide lyase family 24. Kinetic analysis showed that FoRham1 has the highest catalytic efficiency for the substrate α-l-rhamnose-(1→4)-d-glucuronic acid. The crystal structures of ligand-free and α-l-rhamnose-(1→4)-d-glucuronic acid –bound FoRham1 were determined, and the active site was identified on the anterior side of the β-propeller. The three-dimensional structure of the active site and mutagenesis analysis revealed the detailed catalytic mechanism of FoRham1. Our findings offer a new enzymatic tool for the further analysis of the GA carbohydrate structure and for elucidating its physiological functions in plants. Based on these results, we renamed glycoside hydrolase family 145 as a new polysaccharide lyase family 42, in which FoRham1 is included.

The arabinogalactan protein (AGP) family of proteoglycans is present in the extracellular matrix, plasma membrane, and cell walls of a wide variety of plants (1, 2). AGPs are involved in many physiological processes in plants, including the stress response, cell death, cell elongation, intercellular adhesion, and intercellular signal transduction (3). AGPs are composed of type II arabinogalactan, a carbohydrate moiety that typically accounts for more than 90% of the proteoglycan structure, and a core protein rich in hydroxyproline. Type II arabinogalactan has β-1,3-galactan as the main chain and β-1,6-galactooligosaccharide side chains, which are substituted with d-galactose (Gal), l-arabinofuranose (Ara*f*), l-arabinopyranose, d-glucuronic acid (GlcA), 4-*O*-methyl d-glucuronic acid (4-*O*-Me-GlcA), l-rhamnose (Rha), l-fucose, and d-xylose (1, 4, 5). Owing to this complicated structure, the structure–function relationships of AGPs have not yet been elucidated in detail (6).

Gum arabic (GA), a representative AGP, is a sticky exudate from acacia trees. It is produced during stress conditions such as drought and in response to wounds (7). GA has several industrial applications; it is used in the food, cosmetics, and pharmaceutical industries as an emulsifier, stabilizer, and thickener (8). To date, the structure of GA has been analyzed using chemical methods such as methylation and NMR analysis (9, 10); however, its detailed structure has not yet been elucidated, because it is a complex branched polysaccharide. A schematic structure of GA is shown in Figure 1. Enzymes that act on specific glycosidic linkages of carbohydrates are useful tools for elucidating the structure of these carbohydrates and modifying their physical properties. Currently, studies on GA-degrading enzymes are incomplete. There are no reports of complete degradation of GA by enzymes.

**Figure 1:**
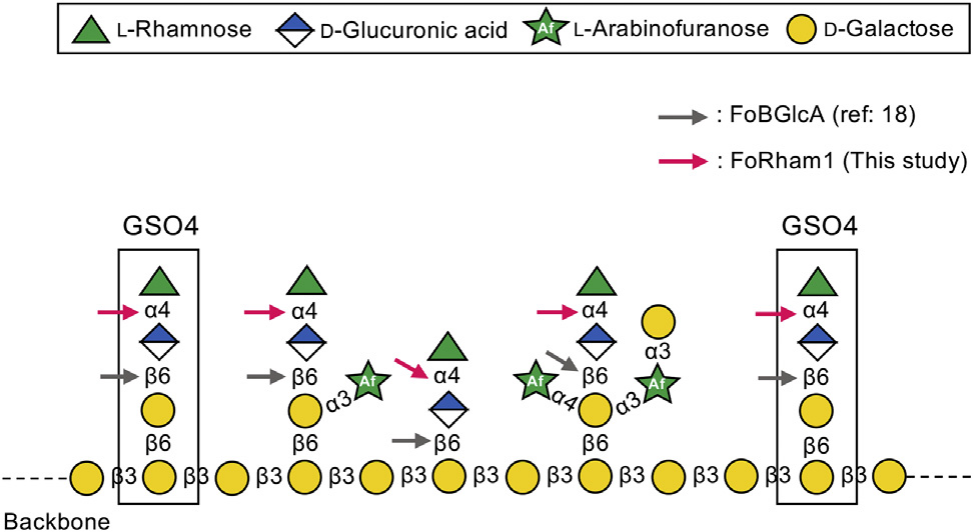
**Schematic structure of GA.** GA has a β-1,3-galactan backbone to which various sugars such as Rha, GlcA, Ara*f*, and Gal are bound as the side chains. The cleavage sites of the two enzymes that play an important role in this study are indicated by *arrows*. GA, gum arabic.

The nonreducing ends of GA side chains are often capped with Rha. Therefore, enzymes that can target and release Rha from GA would be valuable tools for elucidating the detailed structure and function of GA. In the Carbohydrate-Active enZYmes database (11), GA-specific rhamnosidic enzymes are classified into glycoside hydrolases (GHs), belonging to GH145, and polysaccharide lyases (PLs), belonging to PL27. To date, these enzymes have only been reported from bacteria. The GH145 α-rhamnosidase (BT3686) from *Bacteroides thetaiotaomicron* can release Rha from GA but does not act on *p*-nitrophenyl α-l-rhamnopyranoside (*p*NP-α-l-Rha), rhamnogalacturonan (RG)-I, or RG-II (12). BT3686 specifically hydrolyzes the glycosidic linkages of α-l-rhamnose-(1→4)-d-glucuronic acid (Rha–GlcA) present in GA side chains to release Rha through an anomer-retaining mechanism. The catalytic domain of BT3686 consists of a seven-bladed β-propeller fold. The position of the active site is on the “posterior” surface of the β-propeller, which is reversed compared with that of many other β-propeller enzymes. His48 is considered to be a catalytic residue in this enzyme, but the detailed mechanism of the catalysis is unknown (12). Intriguingly, the catalytic histidine is only conserved in ~70% sequences of the homologs of BT3686, and a part of the current GH145 member (*e.g.*, BACCELL_00856) did not show any hydrolytic activity because of a mutation at this residue, typically from H to Q. Three PL27 enzymes from *Bacteroides cellulosilyticus* (BACCELL_00875), *Bacteroides finegoldii* (BACFIN_07013), and *B. thetaiotaomicron* (BT0263) have been reported to release Rha from GA (13). The mechanism involves the desaturation of GlcA at subsite +1 based on a β-elimination reaction. The domain structure of PL27 enzymes comprises an N-terminal β-sandwich structure and a C-terminal (α/α)_6_-barrel structure (13).

Previously, we isolated, cloned, and characterized various GA-degrading enzymes from *Fusarium oxysporum* 12S, a phytopathogenic fungus that can grow using GA as the sole carbon source (14, 15, 16, 17, 18). In addition to these enzymes, we recently observed that Rha was released from GA by the culture supernatant of this fungus.

Here, we isolated the Rha-releasing enzyme (designated FoRham1) specific to GA from the *F. oxysporum* 12S culture supernatant, cloned, and expressed the gene encoding FoRham1 in *Pichia pastoris*, determined the enzymatic properties of FoRham1 using the recombinant enzyme, and performed X-ray crystallography of FoRham1. Through these analyses, we clarified the structure–function relationship of FoRham1, which was given a new family as PL42. The enzyme should offer a foundation for further studies on carbohydrate-related enzymes, leading to new insights.

## Results and discussion

### Purification and internal amino acid sequences of native FoRham1

When the culture supernatant of *F. oxysporum* 12S, which was prepared according to the previous article (17), was reacted with GA, Rha was detected using high-performance anion-exchange chromatography with a pulsed amperometric detector (HPAEC-PAD). We named the Rha-releasing enzyme FoRham1. Native FoRham1 was purified from the culture supernatant (2.8 l) using four columns: Toyopearl DEAE, MonoQ, Resource PHE, and Superdex 200, based on the Rha-releasing activity from GA. SDS-PAGE analysis of the purified enzyme showed a single protein band with a molecular mass of 48 kDa (Fig. S1*A*). Analysis of LC/ion trap (IT)/TOF mass spectrometry detected three peptide sequences of native FoRham1: IGQSGSGDSYIHR, MVNQEGQLIDTK, and SLAEIPNTSTEPLFDK. Information on these peptides is summarized in Table S1. A Mascot search (http://www.matrixscience.com/) identified the protein as matching three peptides with 100% amino acid identity: an uncharacterized protein, FFUJ_14848, from *Fusarium fujikuroi* IMI 58289 (UniProtKB/TrEMBL: S0DZH9). This protein was classified as belonging to GH145 with a Probability-Based Mowse Score of 116 (Figshare: 10.6084/m9.figshare.14634492). Detailed enzymatic properties were determined using the recombinant enzyme, considering the potential for contamination by related enzymes.

### Nucleotide sequence and amino acid analysis of the gene encoding FoRham1

FFUJ_14848, therefore, shows high amino acid sequence similarity with FGRAMPH1_01T21617 from *Fusarium graminearum* PH-1 and FVRRES_10681 from *Fusarium venenatum* A3/5, which belong to GH145. Degenerate primers were prepared based on the nucleotide sequences of the aforementioned three genes. The mature *Forham1* gene was PCR amplified using the primers and complementary DNA of *F. oxysporum* 12S as a template, and the nucleotide and amino acid sequences were determined (DDBJ/EMBL/GenBank accession number: LC617219) (Fig. S2). The length of the mature *Forham1* was 1257 bp, coding for 419 amino acids. The isoelectric point was calculated to be 5.29, and the molecular weight was 47,176, similar to the molecular mass (48 kDa) of a native FoRham1. Two asparagine residues (positions 247 and 377) were identified as *N*-glycosylation sites.

### Sequence comparison and phylogenetic tree analysis

Comparison of the amino acid sequences of FoRham1 with uncharacterized proteins including FFUJ_14848, FGRAMPH1_01T21617, and FVRRES_10681 revealed 95, 90, and 90% identity, respectively. The enzymatic properties of BT3686 from *B. thetaiotaomicron* VPI-5482, BACCELL_00856 from *B. cellulosilyticus* DSM 14838, and HMPREF9455_02360 from *Dysgonomonas gadei* American Type Culture Collection BAA-286, which belong to GH145, have been determined (12). BT3686 shows activity on GA and Rha–GlcA, but not on *p*NP-α-l-Rha, and HMPREF9455_02360 and the Q48H mutant of BACCELL_00856 show activity against GA. However, FoRham1 showed low amino acid sequence identity of 20 to 23% to the three proteins. An amino acid sequence alignment revealed that FoRham1 did not conserve the catalytic histidine of GH145 (His48 in BT3686) (Fig. S2). Pfam analysis (http://pfam.xfam.org/) revealed that FoRham1 is a member of the BNR 4 family (PF15892), which has a bacterial neuraminidase repeat sequence motif (SxDxGxTW) (Fig. S2; *red line*). The repeat sequence has been confirmed in many carbohydrate hydrolases and extracellular proteins with unknown functions.

**Figure 2:**
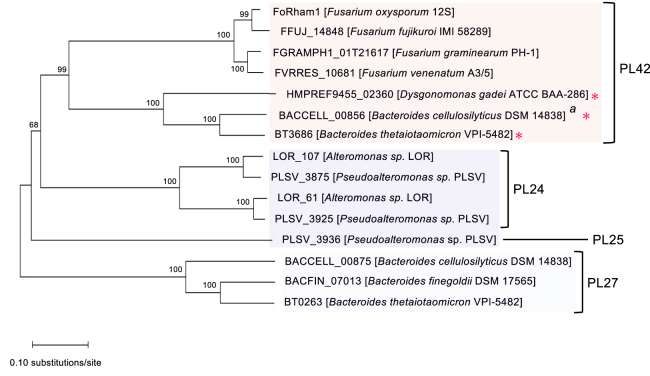
**Phylogenetic relationships between FoRham1 and other enzymes (proteins).** A phylogenetic tree was constructed with the program MEGA X using the algorithm MUSCLE. The neighbor-joining algorithm method was used. A bootstrap analysis of 1000 replications was performed, and the values (%) for the major branches are shown. Proteins indicated by *asterisks* were formerly classified in GH145 and have α-rhamnosidase (hydrolase) activity. ^a^Q48H mutant shows α-rhamnosidase activity.

A phylogenetic tree analysis was performed using the amino acid sequences of FoRham1 and the related proteins belonging to the GH145, PL24, PL27, and PL25 families, which have a structure similar to the β-propeller fold of GH145 (Fig. 2). FoRham1 is evolutionarily distant from PL24, PL25, and PL27. FoRham1 and three *Fusarium* GH145 proteins of unknown function belonged to the same cluster and were classified into a neighboring cluster by the three GH145 α-l-rhamnosidases (Fig. 2).

### Expression and purification of recombinant enzymes

The mature *Forham1* gene was linked to the plasmid pPICZαA and expressed in *P. pastoris* as a fusion protein with a 6× histidine tag and c-Myc tag. The recombinant FoRham1 was purified from the culture supernatant in two steps, using nickel-affinity and anion-exchange chromatographies. The FoRham1 solution after immobilized-metal affinity chromatography purification was contaminated with proteins with slightly different electrophoretic mobility (probably partially modified or degraded FoRham1) (Fig. S1*B*). The contaminants were removed by anion exchange chromatography (Fig. S1*B*, *black lines*). On the SDS-PAGE gel, FoRham1 migrated as a single band with an estimated molecular mass of ~50 kDa (Fig. S1*C*), consistent with the molecular mass of the native enzyme (48 kDa). Size-exclusion chromatography using Superdex 200 provided an estimated molecular mass of 55 kDa for the nondenatured recombinant FoRham1 protein, suggesting that it is a monomer.

### Enzymatic characteristics of FoRham1

The specific activity of the enzyme was 0.3 U/mg using GA as the substrate. Optimal conditions for enzyme reaction were pH 7.0 and 30 °C. The enzyme was stable up to 40 °C and in the pH range of 7.0 to 10.4 (Fig. S3). The substrate specificity of FoRham1 was examined using various substrates (Table 1). The enzyme was highly active against Rha–GlcA and GA but showed trace activity against ulvan and larch wood arabinogalactan (LWAG) and was undetectable against RG-I oligosaccharides, which have Rha residues at their nonreducing end, *p*NP-α-l-Rha, and quercetin 3-*O*-(6-*O*-α-l-rhamnopyranosyl)-β-d-glucopyranoside (rutin).

**Table 1:** Relative activity of FoRham1 toward various substrates

Substrate	Relative activity (%)
Rha–GlcA	100 ± 0
GA	42 ± 0.6
Ulvan	Trace
LWAG	Trace
RG-I oligosaccharides	ND
*p*NP-α-l-Rha	ND
Rutin	ND

Abbreviation: ND, not detectable.

Enzyme activity was measured using 0.1% polysaccharides and 0.5 mM oligosaccharides as substrates in 20 mM Hepes–NaOH (pH 7.0). The enzyme units used for the measurement were 0.05 mU for GA and Rha–GlcA and 7.5 mU for other substrates. The reaction was performed at 30 °C for 15 min to 16 h. After boiling the mixture for 5 min, the released Rha was quantified by HPAEC-PAD. All experiments were performed in triplicate (n = 3) and expressed as mean ± SE. Trace < 0.05%.

**Figure 3:**
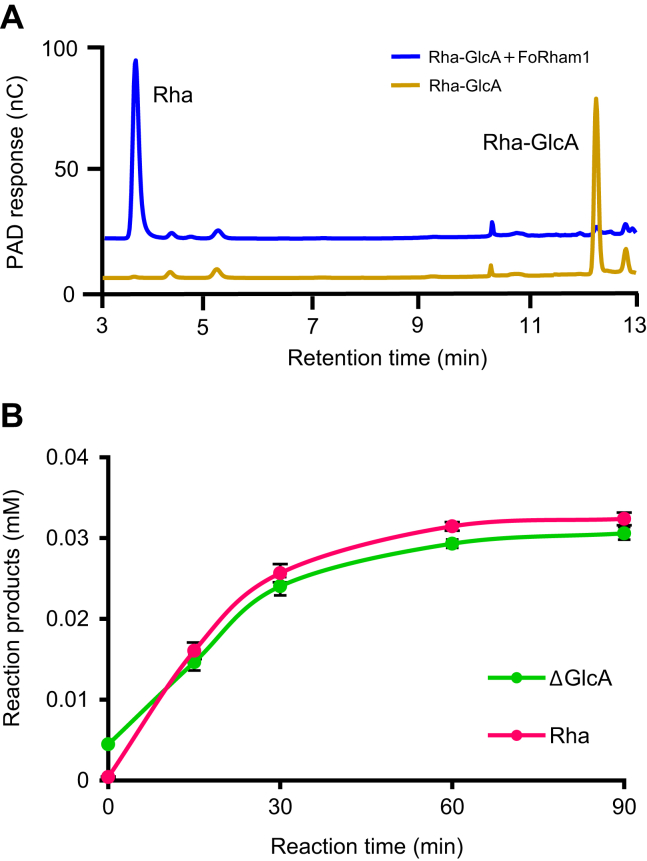
**Analysis of enzymatic degradation products.***A*, detection of the reaction product of FoRham1 for Rha–GlcA. *Gray line*, before the reaction; *pink line*, after the reaction. *B*, quantification of the reaction product of FoRham1 for GA. *Pink line*, Rha; *blue line*, ΔGlcA. Concentration of ΔGlcA was determined by measuring the absorbance of the reaction mixture at 235 nm. The molar extinction coefficient of ΔGlcA at 235 nm is 4800 M^−1^ cm^−1^. All experiments were performed in triplicate (*n* = 3) and expressed as mean ± SD.

**Figure 4:**
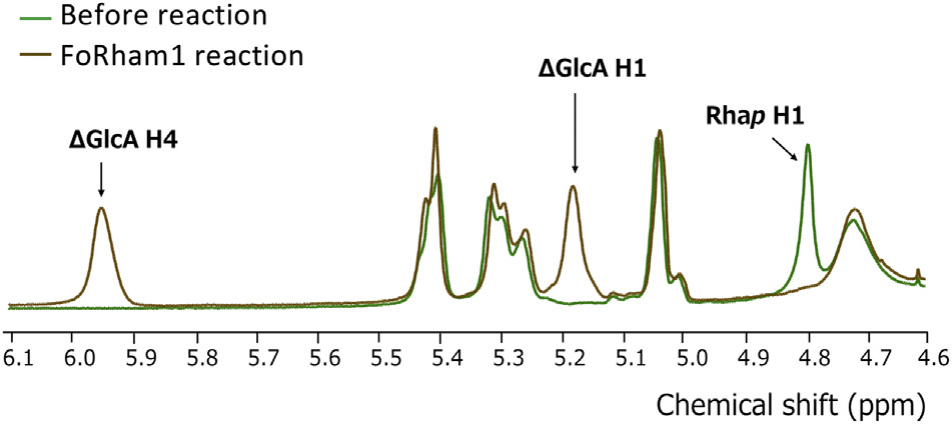
^**1**^**H-NMR analysis of GA treatment with FoRham1.** Experimental conditions are described in the text. *Green line*, before the reaction; *brown line*, after the reaction. GA, gum arabic.

Since GA has an Rha–GlcA structure at the end of the side chains (10, 18), FoRham1 seemed to act specifically on Rha residues bound to GlcA by α-1,4-linkages. GH145 α-l-rhamnosidase BT3686 also shows activity on GA and Rha–GlcA, but not on *p*NP-α-l-Rha, RG-I, or RG-II (12). However, when the product of Rha–GlcA degradation by FoRham1 was analyzed using HPAEC-PAD, only Rha was detected (Fig. 3*A*), suggesting that the enzyme is not a hydrolase but a lyase. PLs are enzymes that cleave uronic acid–containing carbohydrates by a β-elimination reaction, producing an unsaturated sugar moiety at the nonreducing end of the cleavage site. When FoRham1 degrades Rha–GlcA, it generates Rha and Δ4,5-unsaturated GlcA (ΔGlcA), but the latter pyranose ring can be nonenzymatically opened and converted to 4-deoxy-l-threo-5-hexosulose-uronate. Therefore, it was considered that the ΔGlcA product was not detected by HPAEC-PAD. The amounts of Rha and ΔGlcA produced from GA by FoRham1 were measured, confirming that these products were produced in equimolar amounts (Fig. 3*B*). GA was treated with FoRham1, and GAs before and after the enzyme reaction were analyzed using ^1^H-NMR. After the enzyme treatment, the signal attributed to Rha H-1 (4.797 ppm) disappeared, and the signal attributed to ΔGlcA H-1 (5.177 ppm) and H-4 (5.947 ppm) appeared (Fig. 4). The three signals were assigned according to previous reports (19, 20, 21).

The results described previously indicated that FoRham1 was an l-rhamnose-α-1,4-d-glucuronic acid lyase. At present, this kind of enzyme activity has been observed in only three proteins: BT0263 from *B. thetaiotaomicron* VPI-5482, BACCELL_00875 from *B. cellulosilyticus* DSM 14838, and BACFIN_07013 from *B. finegoldii* DSM 17565, all of which are classified into PL27 (13). However, amino acid sequence identities between FoRham1 and the aforementioned three proteins were quite low (3–5%), indicating that FoRham1 should not be classified into PL27.

**Figure 5:**
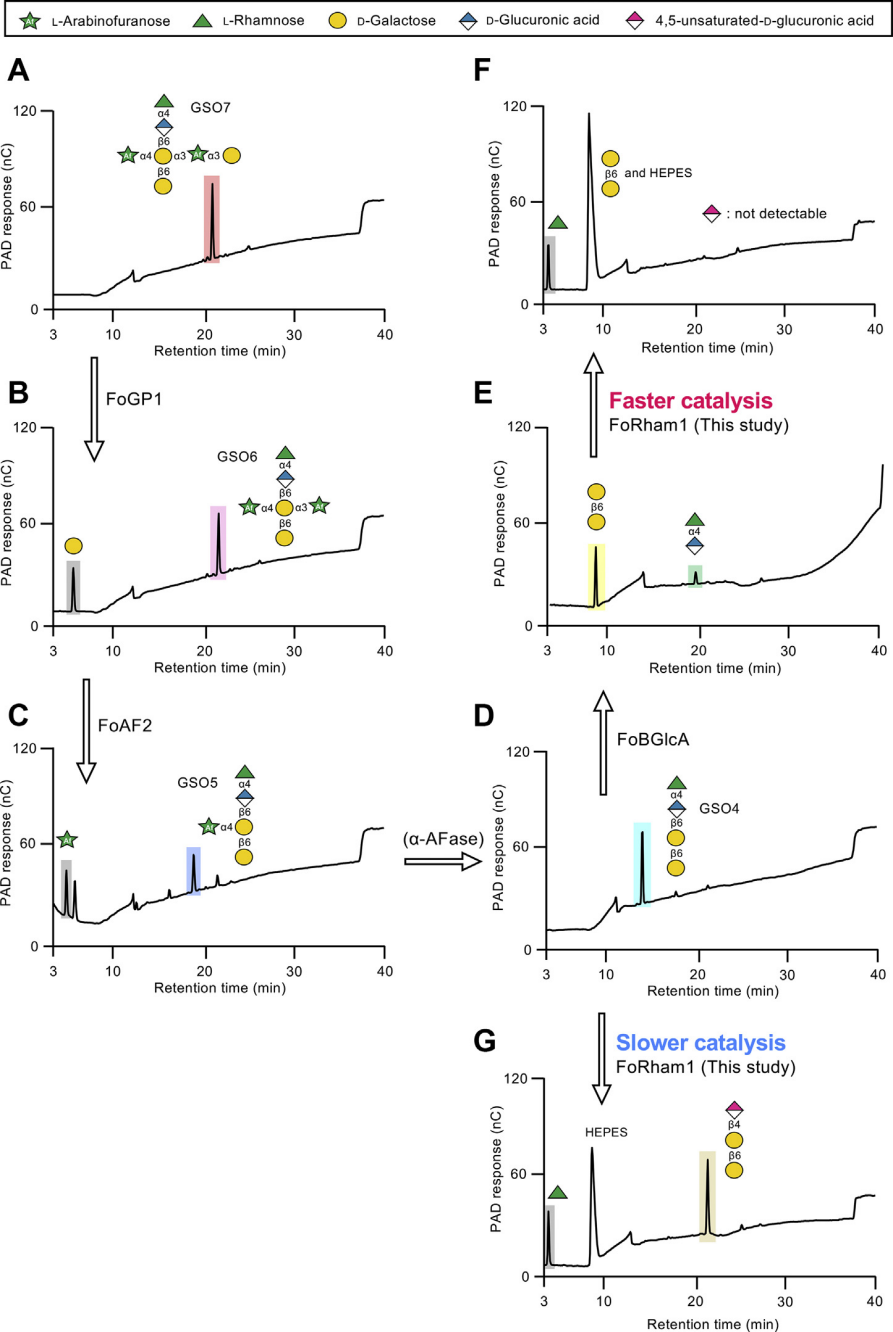
**Putative degradation pathway of GSO7 in GA by *F. oxysporum* 12S based on enzyme activity.***A*, purified GSO7; *B*, GSO7 was digested with FoGP1; *C*, GSO6 was digested with FoAF2; *D*, purified GSO4; *E*, GSO4 was digested with FoBGlcA; *F*, Rha–GlcA was digested with FoRham1; *G*, GSO4 was digested with FoRham1. GSOs were analyzed by HPAEC-PAD. GA, gum arabic; GSO, oligosaccharide derived from side chains of gum arabic; HPAEC-PAD, high-performance anion-exchange chromatography with a pulsed amperometric detector.

Using the enzymatic properties of FoRham1, the binding position of Rha in GA was examined. When GA was thoroughly digested with FoRham1, approximately 95% of Rha in GA was released, indicating that most Rha residues were present at the nonreducing ends of the side chains, and were bound to GlcA by α-1,4 linkages in GA. We evaluated whether FoRham1 prefers a polymer or an oligomer. In addition to natural GA, GA degraded by GH79 4-*O*-α-l-rhamnosyl-β-d-glucuronidase (FoBGlcA; GenBank accession number: LC534636) (18), which we termed GA-RG, was used as the substrate. FoBGlcA hydrolyzes the β-1,6-glucuronic linkage of GA and releases Rha–GlcA (Fig. 1). The kinetic parameters toward GA and GA-RG were measured (Table 2 and Fig. S4). FoRham1 had a higher affinity and catalytic efficiency for GA-RG (probably for the Rha–GlcA disaccharide released from GA) than natural GA and was found to prefer a low molecular mass substrate. When the kinetics of FoRham1 for Rha–GlcA and the oligosaccharide derived from side chains of GA (GSO4), which is a tetrasaccharide of Rha–GlcA bound to β-1,6-galactobiose (Fig. 1), were determined, the catalytic efficiency for Rha–GlcA was five times higher than that of GSO4 (Table 3 and Fig. S4). The specific activity of FoRham1 for oligosaccharides (GSO5−7) having different side chains bonded to GSO4 was determined. The structures of these oligosaccharides are shown in Figure 5. The enzyme activity of FoRham1 did not change much irrespective of the type of side chain (Table 4). These results suggest that FoRham1 is an enzyme that efficiently degrades Rha–GlcA released from GA with FoBGlcA.

**Table 2:** Kinetic parameters of the FoRham1 toward polysaccharides

Substrate	*K*_m_ (mg/ml)	*k*_cat_ (s^−1^)	*k*_cat_/*K*_m_ (s^−1^ mg/ml^−1^)
GA	41.3 ± 5.9	39.6 ± 3.2	0.96
GA-RG	14.4 ± 1.6	63.4 ± 2.9	4.4

Enzyme kinetics were determined by incubating reaction mixtures consisting of substrates (0.4–5%) in 50 mM Hepes–NaOH (pH 7.0) and FoRham1 (20 nM) at 30 °C for 30 min. The enzyme was then inactivated by boiling, and the sugar released was quantitated by HPAEC-PAD. *K*_m_ and *k*_cat_ were calculated for eight substrate concentrations using a nonlinear regression plot (n = 3).

**Table 3:** Kinetic parameters of the FoRham1 toward oligosaccharides

Substrate	*K*_m_ (mM)	*k*_cat_ (s^−1^)	*k*_cat_/*K*_m_ (s^−1^ mM^−1^)
Rha–GlcA	2.8 ± 0.2	95.5 ± 2.6	34.1
GSO4	5.2 ± 0.5	37.3 ± 1.4	7.2

Enzyme kinetics were determined by incubating reaction mixtures consisting of substrates (0.1–20 mM) in 50 mM Hepes–NaOH (pH 7.0) and FoRham1 (20 nM) at 30 °C for 30 min. The enzyme was then inactivated by boiling, and the sugar released was quantitated by HPAEC-PAD. *K*_m_ and *k*_cat_ were calculated for eight substrate concentrations using a nonlinear regression plot (n = 3).

**Table 4:** Specific activities of the FoRham1 toward oligosaccharides

Substrate	Specific activity (μmol/min/mg)
Rha–GlcA	8.10 ± 1.07
GSO4	1.25 ± 0.18
GSO5	1.32 ± 0.13
GSO6	1.03 ± 0.03
GSO7	0.97 ± 0.06

FoRham1 (0.2 μM) was incubated with Rha–GlcA and GSO4−7 (1 mM) in 50 mM Hepes–NaOH (pH 7.0) at 30 °C for 5 min. The released Rha was quantified by HPAEC-PAD. All experiments were performed in triplicate (n = 3) and expressed as mean ± SE.

Figure 5 shows the putative degradation pathway of the complex GSO7 side chain in the GA by *F. oxysporum*. GSO7 (Fig. 5*A*) is degraded to GSO5 by the successive actions of GH27 α-galactosidase (FoGP1; (17)) and GH54 α-l-arabinofuranosidase (FoAF2: unpublished data) (Fig. 5, *B* and *C*). In turn, GSO5 may be degraded to GSO4 by α-l-arabinofuranosidase (Fig. 5*D*). This enzyme has not yet been isolated from *F. oxysporum*. GH79 4-*O*-α-l-rhamnosyl-β-d-glucuronidase (FoBGlcA; (18)) degrades GSO4 to Rha–GlcA and β-1,6-galactobiose (Fig. 5*E*). Rha–GlcA is degraded to Rha and ΔGlcA by FoRham1, as described in this study (Fig. 5*F*). Based on the kinetic parameters and the results of enzymatic activity against GSOs, the catalytic reaction of FoRham1 against GSO4, another possible pathway, was suggested to be unfavorable (Fig. 5*G*). The discovery of a second α-l-arabinofuranosidase is a future research objective.

### Structure determination and overall structure

**Figure 6:**
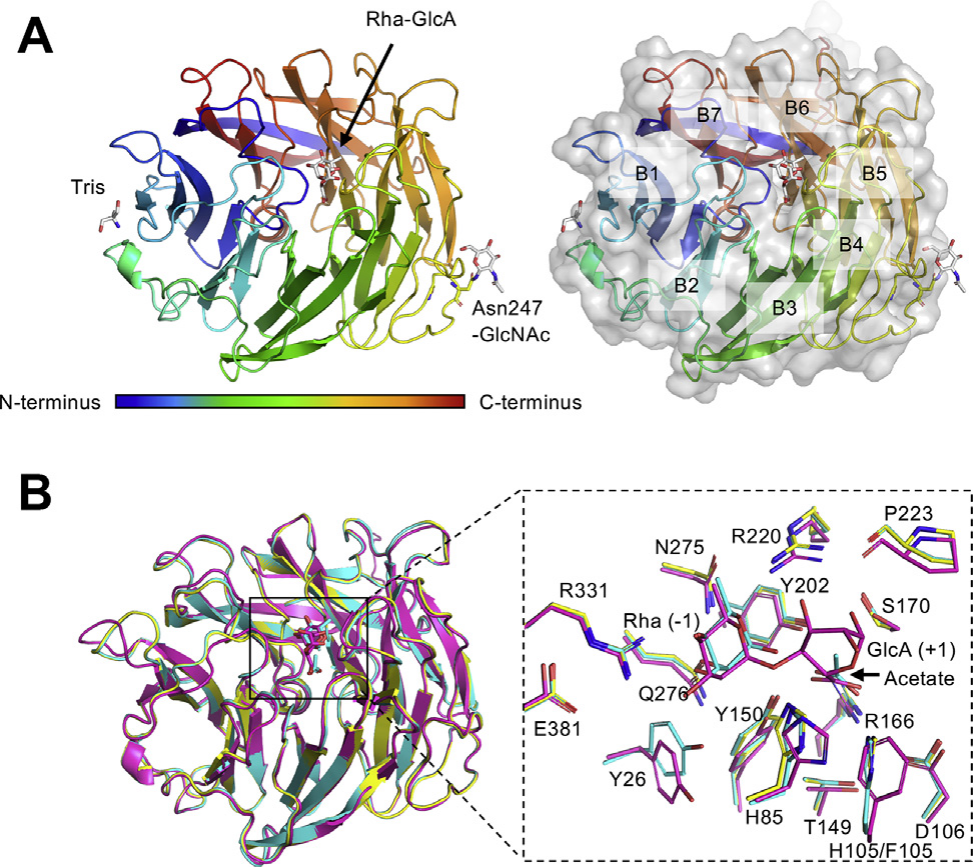
**Overall crystal structure of FoRham1.***A*, the overall structure of FoRham1 (H105F mutant, *rainbow color* shown in *blue* from the N terminus to *red* at the C terminus) complexed with Rha–GlcA is shown as *ribbon* (*left*) and *surface* (*right*) models. *N*-glycan and the ligands are shown as *gray sticks*. The seven blades (B1–B7) are assigned based on the β-propeller structure (34). *B*, superimposition of the ligand-free (*yellow*), Rha complex (*cyan*), and H105F Rha–GlcA complex (*magenta*) structures at the catalytic site.

WT FoRham1 was labeled with selenomethionine (FoRham1 SeMet), and the structure was resolved *via* the single-wavelength anomalous dispersion method using the selenium atom (Table 5). Attempts at molecular replacement using the crystal structure of GH145 BT3686 as a search model failed, owing to low protein structure similarity with available templates (<30% amino acid sequence identity). The structures of FoRham1 WT (ligand-free form), the reaction product (Rha) complex, and the complex with its substrate (Rha–GlcA) were determined at resolutions of 1.05, 1.4, and 2.42 Å, respectively (Table 5). The Rha–GlcA complex crystals were prepared using an inactive H105F mutant (described later). The crystals contained one protein molecule in an asymmetric unit. Electron densities of the C-terminal 6× histidine and c-Myc tags were not observed for the WT protein structures and not modeled. The Rha–GlcA (H105F) structure forms intermolecular interactions between a crystallographic symmetry pair through the C-terminal tag region (Fig. S5). The structure of FoRham1 comprises a seven-bladed β-propeller domain, and the Rha–GlcA is bound to the cleft at the center of the anterior side (Fig. 6*A*). The catalytic site is flanked by loops of the β-propeller (blades 1, 3, 4, and 6). The Cα RMSD value between FoRham1 structures of the ligand-free and Rha–GlcA complexes is 0.378 Å, indicating that there is no conformational change on the substrate binding (Fig. 6*B*, *left*). However, slight variations in the amino acid residues at the catalytic site were observed (Fig. 6*B*, *right*).

**Table 5:** Data collection and refinement statistics

Dataset	SeMet	WT	WT Rha	H105F Rha–GlcA	N247A
Data collection					
Beamline	BL-5A	BL-5A	NW12A	BL-5A	BL-5A
Wavelength (Å)	0.9789	1.0000	1.0000	1.0000	1.0000
Space group	*P*2_1_2_1_2_1_	*P*2_1_2_1_2_1_	*P*2_1_2_1_2_1_	*C*222	*P*2_1_2_1_2_1_
Unit cell (Å)	*a = 58.3*	*a = 58.1*	*a = 55.2*	*a = 127.2*	*a = 57.6*
	*b = 65.4*	*b = 65.3*	*b = 65.0*	*b = 190.1*	*b = 65.3*
	*c = 107.4*	*c = 107.0*	*c = 107.7*	*c = 80.1*	*c = 107.3*
Resolution (Å)^a^	50.0–1.60 (1.63–1.60)	50.0–1.05 (1.07–1.05)	50.0–1.40 (1.42–1.40)	50.0–2.42 (2.51–2.42)	50.0–1.40 (1.42–1.40)
Total reflections	1,408,921	1,173,670	512,548	253,105	457,776
Unique reflections^a^	104,551 (5271)	190,044 (9391)	77,482 (3830)	37,476 (3884)	75,319 (2667)
Completeness (%)^a^	99.9 (99.8)	99.9 (99.9)	98.9 (97.5)	100 (99.9)	94.2 (68.0)
Redundancy^a^	13.5 (13.0)	6.2 (5.4)	6.6 (6.7)	6.8 (6.9)	6.1 (5.6)
Mean I/σ(I)^a^	36.7 (3.6)	39.4 (2.0)	14.0 (2.8)	17.7 (1.9)	24.5 (1.3)
*R*_merge_ (%)^a^	7.9 (70.2)	5.0 (71.6)	6.9 (55.3)	7.3 (96.0)	7.9 (83.3)
CC_1/2_^a^	1.000 (0.897)	0.999 (0.931)	0.999 (0.882)	0.997 (0.875)	1.000 (0.917)
Refinement					
Resolution (Å)		31.30–1.05	41.52–1.40	46.3–2.42	43.23–1.40
No. of reflections		179,860	73,520	35,629	71,452
*R*_work_/*R*_free_ (%)		15.0/17.0	18.8/21.5	20.7/24.2	16.6/19.2
Number of atoms		4018	3654	3665	3869
RMSD from ideal values					
Bond lengths (Å)		0.023	0.014	0.003	0.011
Bond angles (°)		2.11	1.946	1.347	1.581
Average B-factor (Å2)					
Protein		35.1	14.9	55.2	23.5
Ligand		28.6	30.6	69.6	23.1
Solvent		26.8	17.7	52.2	34.4
Ramachandran plot (%)					
Favored		96.9	95.2	94.7	96.4
Allowed		3.1	3.8	4.6	3.6
Outlier		0	0	0.7	0
Protein Data Bank code		7ESK	7ESM	7ESN	7ESL

a Values in parentheses are for the highest resolution shell.

The protein samples used for crystallization of the WT and H105F were not treated by endo-β-*N*-acetylglucosaminidase or peptide:*N*-glycanase. Therefore, Man-α-1,3-Man-β-1,4-GlcNAc-β-1,4-GlcNAc-β-1-Asn was observed at one of the two potential *N*-glycosylation sites of the WT and Rha complex structures (Asn247; Fig. S6), whereas a GlcNAc residue was observed at this site in the Rha–GlcA complex (Fig. 6*A*). *N*-glycosylation at Asn377 was not observed. Recombinant enzymes that were heterologously expressed by the secreting system of *P. pastoris* are often glycosylated, and high-mannose type *N*-glycan was observed in protein crystal structures (22, 23, 24, 25). To investigate the effect of *N*-glycosylation on the crystal structure, we crystallized the N247A mutant as an *N*-glycan–deficient form. The crystal of the N247A mutant was isomorphous with the WT protein crystals (Table 5), and their crystal structures are virtually the same (Cα RMSD = 0.077 Å). This result revealed that N-glycosylation did not substantially affect the three-dimensional structure of FoRham1.

### Ligand-binding site

**Figure 7:**
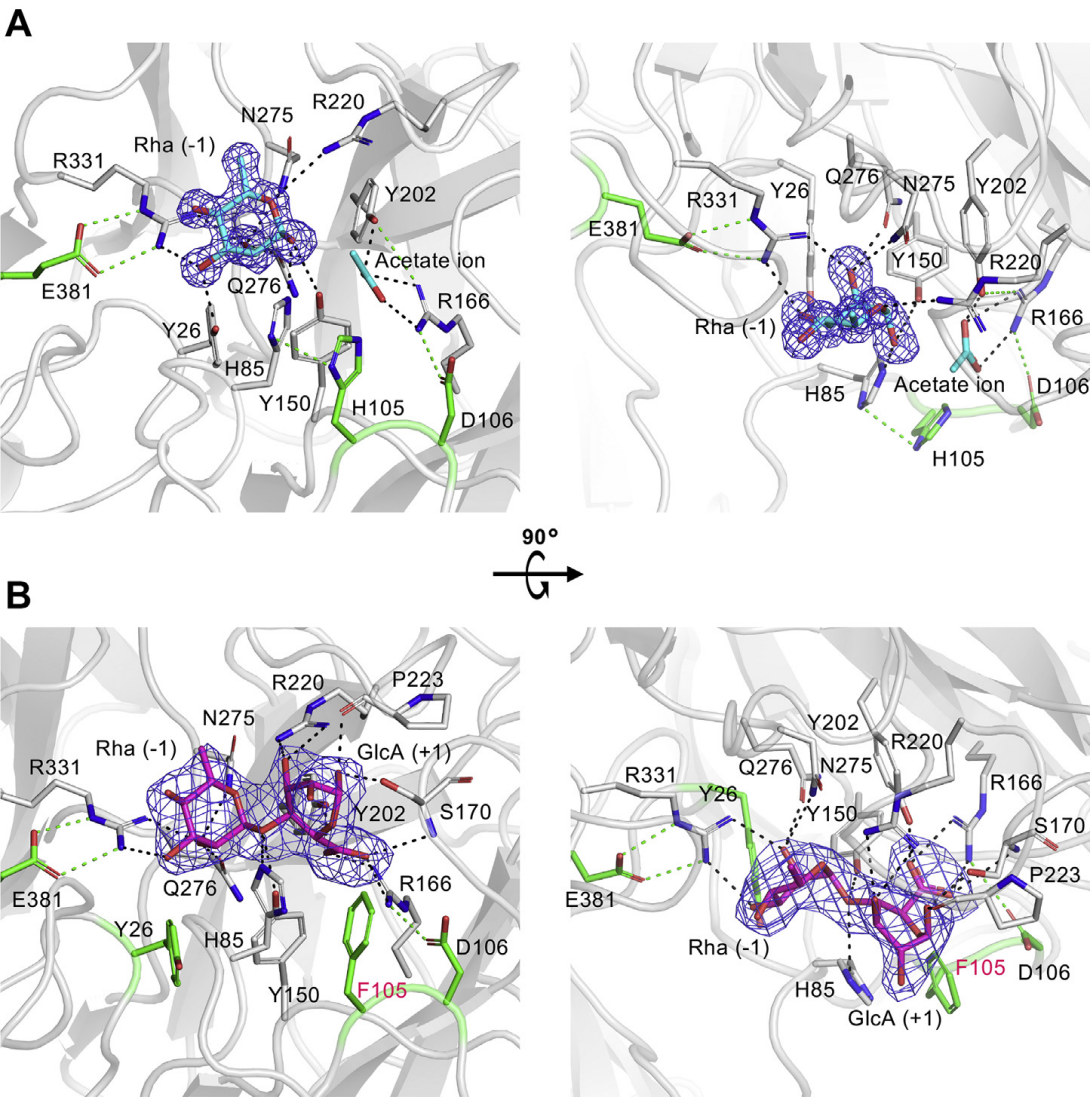
**Ligand-binding site.** The side chains interacting with the ligands and the neighboring side chains are colored in *gray* and *green*, respectively. Hydrogen bonds (<3.5 Å) between the ligand and the protein and between the protein residues are shown as *gray* and *green dotted lines*, respectively. *A*, the ligand-binding site of the Rha complex. The Rha and acetate ion are shown as *cyan sticks*. The *mF*_o_–*F*_c_ omit electron density map (*blue mesh* at 3.0 σ) of Rha is shown. *B*, the ligand-binding site of the H105F Rha–GlcA complex. Rha–GlcA is shown as *magenta sticks*. The *mF*_o_–*F*_c_ omit electron density map (*blue mesh* at 3.0 σ) of Rha–GlcA is shown.

**Figure 8:**
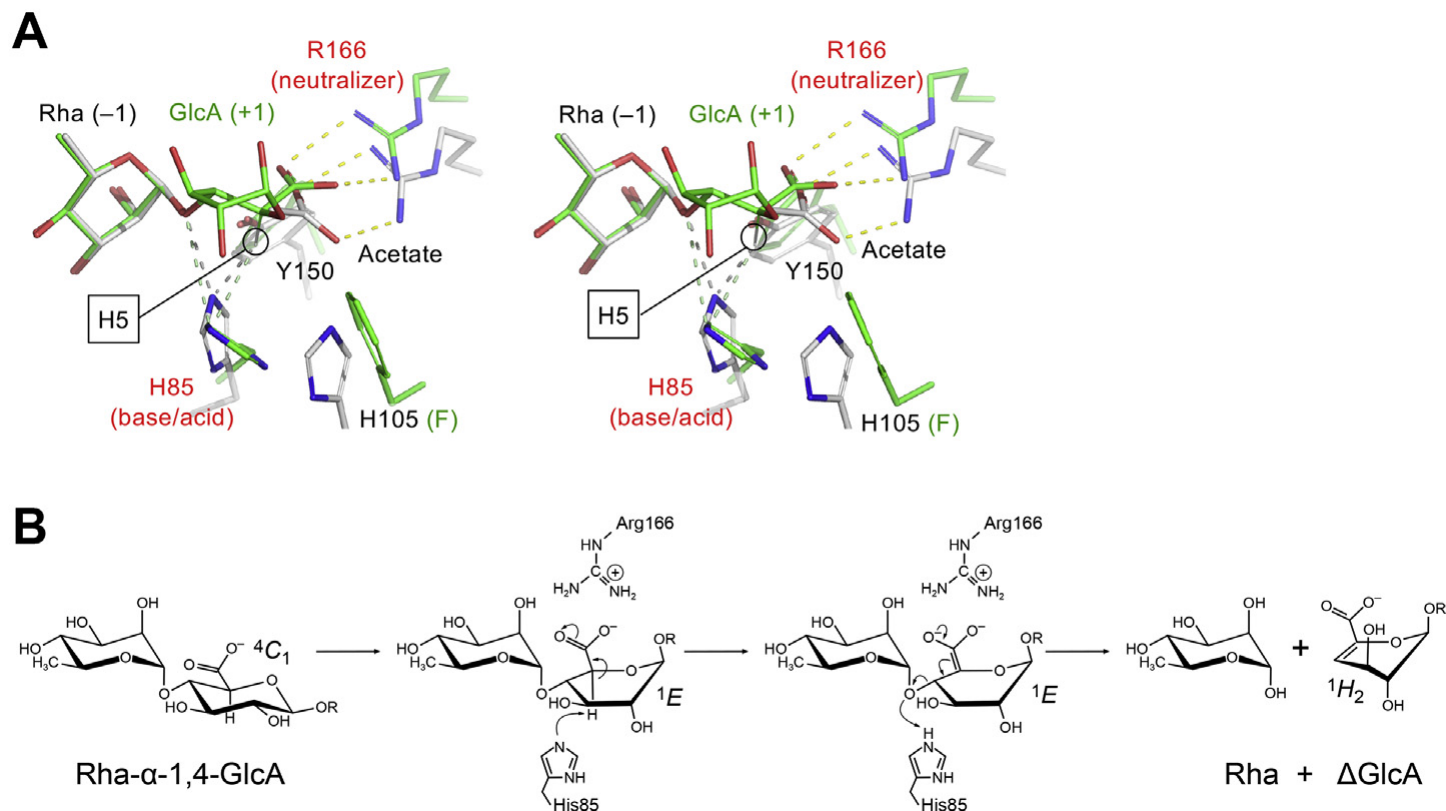
**Interactions in the catalytic area and proposed catalytic mechanism of FoRham1.***A*, stereoview of the Rha–GlcA complex (H105F mutant, *green*) and Rha complex (*white*) superimposed by the pyranose ring atoms of Rha. Arg166 forms a charge interaction with the carboxylic acid of GlcA and functions as the neutralizer. His85 is located near to both the GlcA H5 and the glycosidic bond oxygen atoms and is suggested to be the base–acid catalyst. *B*, proposed catalytic mechanism of FoRham1. R = H or another sugar residue.

Figure 7 shows the Rha and Rha–GlcA binding sites of FoRham1. Both ligands were bound to the cleft located on the anterior side. Electron density maps of the ligands were clearly observed (Fig. 7). The GlcA residue of Rha–GlcA adopts a distorted ^1^*E* conformation (Cremer–Pople parameter: *ϕ* = 249.7°, *θ* = 111.3°, and *Q* = 0.619) (26), which is clearly different from the stable ^4^*C*_1_ conformation in solution (Fig. 7*B*). In the Rha complex, Rha and acetate ion are bound at subsite −1 and subsite +1, respectively (Fig. 7*A*). The O-1 atom of Rha forms a hydrogen bond with the side chain of Tyr150 and His85, suggesting that one of them is a possible catalytic acid residue for the *syn*-β-elimination reaction (Fig. 7*A*, discussed later) (27). The His105 side chain formed a hydrogen bond with the His85 side chain, suggesting its involvement in functional assistance or stabilization. An acetate ion is bound at the binding site of the GlcA carboxylate (subsite +1) in the Rha complex structure and forms hydrogen bonds with the Arg166 and Tyr202 side chains (Fig. 7*A*). Tyr26, Asn275, Gln276, and Arg331 form hydrogen bonds with the O-2 and O-3 atoms of Rha, and Glu381 forms a salt bridge with Arg331, which likely helps to fix Arg331 (Fig. 7*A*). The ligand-binding mode of the Rha–GlcA complex of the H105F mutant was similar to that of the Rha complex, although the positions of Tyr26, His85, Phe105 (His105), and Tyr150 differed slightly (Fig. 7*B*). The Arg166 side chain forms bifurcated hydrogen bonds as well as charge interaction with the carboxylic group of GlcA. His85 is located near the C-5 atom of GlcA (Fig. 8*A*), suggesting its catalytic role as the proton acceptor (discussed later). O-1 of GlcA at subsite +1 interacts with the side chain of Ser170 and the carboxyl group of the Pro223 main chain, and the O-3 atom formed a hydrogen bond with Arg220. Therefore, this conformational distortion of GlcA at subsite +1 is caused by hydrogen bonding with these residues, notably Arg220.

### Site-directed mutant analysis

To investigate the catalytic importance of the amino acid residues in the active site of FoRham1, we prepared site-directed mutants and measured their activity toward GA and Rha–GlcA (Table 6). The activities of His85 mutants (H85A and H85F) were not or barely detected, strongly suggesting that this residue plays a crucial role in catalysis. Activities of His105 mutants (H105A and H105F) were also significantly reduced, highlighting its possible catalytic support function. Therefore, we used the H105F mutant to obtain the complex structure with Rha–GlcA (Table 5 and Fig. 7*B*). The activities of the Y150A mutant were not detected or barely detectable, but Y150F exhibited ~1% activity compared with the WT. This result suggested that Tyr150 does not likely to play a primary role in catalysis. S170A and S170T mutants retained 1.4~89% activity, whereas the activities of R220A and R220K mutants were significantly reduced. This result suggested that Arg220 plays a more important role than Ser170 in supporting the catalysis by conformational fixation of GlcA.

**Table 6:** Relative activity of FoRham1 WT and mutants

Enzyme	Relative activity	
	GA	Rha–GlcA
WT	1.0	1.0
H85A	—a	—a
H85F	3.4 × 10^−4^	—a
H105A	3.5 × 10^−4^	—a
H105F	3.8 × 10^−4^	—a
Y150A	5.5 × 10^−4^	—a
Y150F	9.0 × 10^−3^	1.0 × 10^−2^
R166A	1.6 × 10^−3^	2.1 × 10^−2^
R166K	2.1 × 10^−3^	1.9 × 10^−2^
S170A	8.9 × 10^−1^	3.0 × 10^−1^
S170T	3.6 × 10^−2^	1.4 × 10^−2^
R220A	9.0 × 10^−4^	9.8 × 10^−3^
R220K	5.4 × 10^−4^	—a

The relative activities of FoRham1 and the mutant enzymes were analyzed using GA (1%) and Rha–GlcA (1 mM) as the substrate. The enzymes (5 μM) were incubated with each substrate in 50 mM Hepes–NaOH (pH 7.0) at 30 °C for 5 min. The released Rha was quantified by HPAEC-PAD.

a Not detectable.

### Proposed catalytic mechanism of FoRham1

The catalytic mechanisms of PLs can be divided into two types, depending on their requirements for metal ions (27, 28). In the case of metal-dependent PLs, a divalent cation (Ca^2+^ or Mn^2+^) neutralizes the carboxylic group of the uronate and enhances the reactivity of the C-5 proton. In metal-independent PLs, the charge neutralizer is one or several amino acids, which are usually Glu, Gln, Asp, or Asn associated with His, Arg, or Glu (27). The activity of FoRham1 was slightly increased with the addition of metal ions and EDTA, except for Cu^2+^ and Zn^2+^ ions (Fig. S7). In the crystal structure, an electron density peak of Ca^2+^ ion was found in the interior of the β-propeller fold near the posterior side (Fig. S8), but no metal ion peaks were observed in the catalytic site of the anterior side. Here, we propose the catalytic mechanism of FoRham1 based on the crystal structures and mutational analysis (Fig. 8*B*). When the substrate binds to FoRham1, the GlcA moiety changes its pyranose ring conformation because of the interactions with the protein from stable ^4^*C*_1_ to ^1^*E*, as observed in the Rha–GlcA complex structure (Fig. 7*B*). The distorted conformation of GlcA may alleviate the steric hindrance around the H-5, especially with the C-2 hydroxy group. Elimination from d-glucuronide in PLs is generally thought to proceed *via* a stepwise *syn*-elimination pathway, not a concerted *anti-*elimination (27, 29). Arg166 functions as the neutralizer and enhances the reactivity of the C-5. His85 is the sole candidate of the base catalyst that abstracts the labile proton. The Nε atom of His85 in the Rha–GalA complex structure is at 3.0 Å distance from the modeled H-5 atom of GlcA (Fig. 8*A*, *green dotted line*). The apparent p*K*_a_ value of the steep basic rim (~7.5) of the pH–activity profile of FoRham1 also supports the involvement of base catalysis by a histidine (Fig. S3*C*), and this proton abstraction is probably the rate-limiting step. In the second step, lytic cleavage of the O-4:C-4 bond is facilitated by proton donation from a catalytic acid. Based on the crystallographic and mutational analyses (Fig. 7*A* and Table 6), His85 is also suggested to be the catalytic acid. As observed in the crystal structure (Fig. 8*A*), the His85 side chain probably changes its conformation during the two catalytic steps, and His105 may support this base–acid dual function. In the Rha complex structure, the distance of the Nε atom of His85 from the modeled GlcA H-5 and Rha O-1 atoms are 2.2 and 3.0 Å, respectively (Fig. 8*A*, *gray dotted lines*). As to metal-independent *syn*-eliminating PLs, a single residue (Tyr or His) often functions as the base–acid catalyst (13, 28, 30). After the cleavage, Rha and ΔGlcA-containing polymers or monosaccharides are released from the enzyme. ΔGlcA adopts two major half-chair conformations in solution (^2^*H*_1_ or ^1^*H*_2_) (31, 32), and the latter resembles the ^1^*E* conformation of the GlcA moiety bound to FoRham1.

### Structural comparison of FoRham1 with GH145, PL24, and PL25

**Figure 9:**
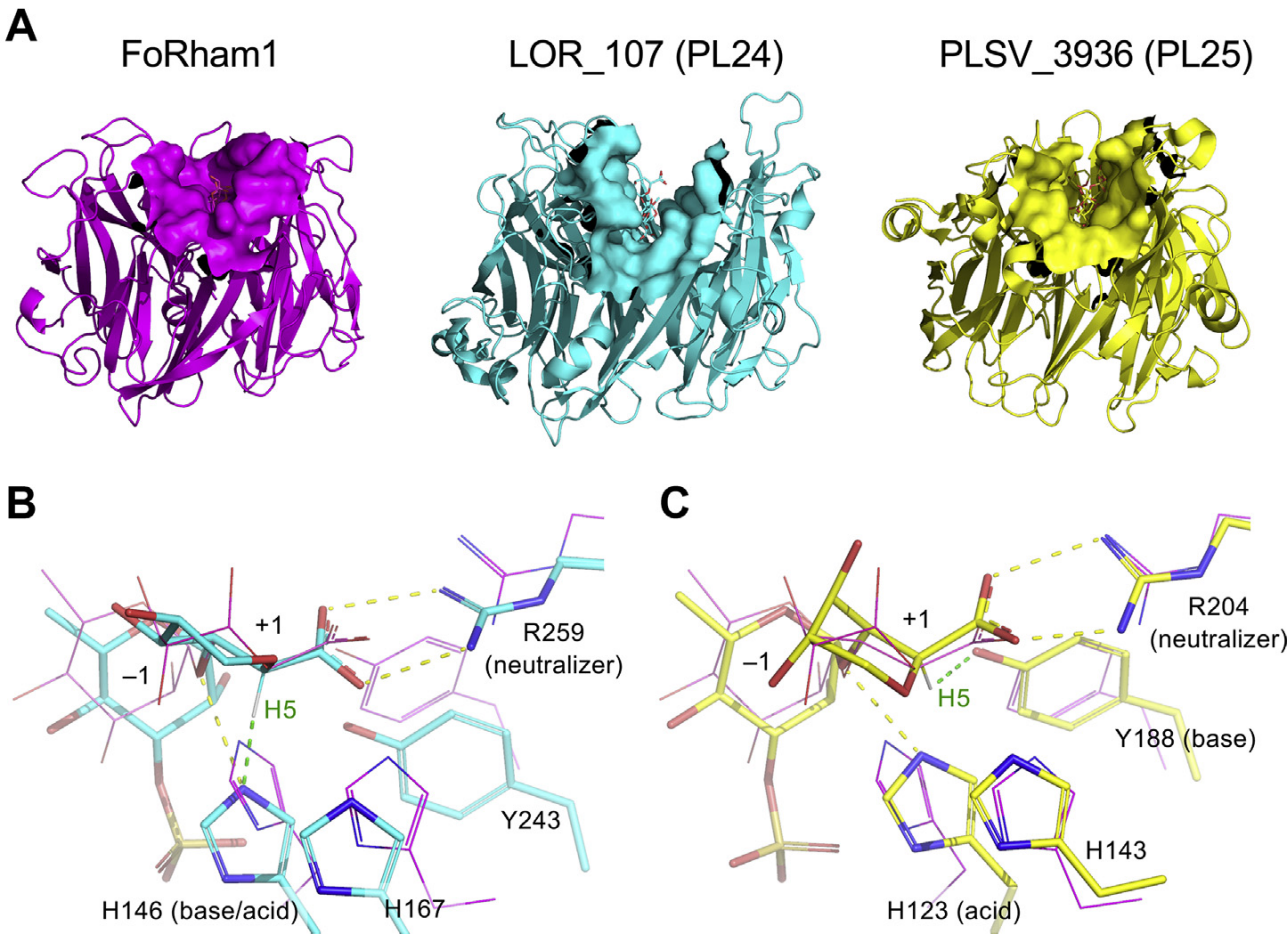
**Comparison with PL24 and PL25.***A*, overall structures of FoRham1 (*left*, *magenta*), PL24 LOR_107 (*middle*, *cyan*; Protein Data Bank [PDB] ID: 6BYT), and PL25 PLSV_3936 (*right*, *yellow*, PDB ID: 5UAS). The bound ligands are shown as *sticks*, and the ligand-binding sites are shown by molecular surface (around 10 Å). *B* and *C*, the catalytic site of LOR_107 (*B*, *cyan*) and PLSV_3936 (*C*, *yellow*; PDB ID: 5UAM for the protein side chains). Hydrogen bonds and the interaction for proton abstraction are shown as *yellow* and *green dotted lines*, respectively. The FoRham1 structure is superimposed (*magenta lines*).

The result of the structural similarity search using the Dali server (33) is shown in Table S2. FoRham1 most closely resembled the four GH145 proteins (Z score > 43) (12). Two ulvan lyases belonging to PL25 (34) and PL24 (30) also showed high structural similarity (Z score > 32), and lectins are moderately similar (Z score < 27). Fig. S9 compares the structures of FoRham1 with representative members of GH145 (BT3686), PL24 (LOR_107), and PL25 (PLSV_3936), illustrating that their seven-bladed β-propeller structures are superimposed well. Figure 9 shows a structural comparison with the two PLs. The catalytic site of exolytic FoRham1 appeared as a shallower pocket than those of LOR_107 and PLSV_3936, which were reported as endo-type ulvan lyase (Fig. 9*A*). In general, the catalytic clefts of endolytic enzymes are deep or penetrating, whereas exolytic enzymes often have shallower pockets as catalytic sites (35, 36). LOR_107 utilizes Arg259 and His146 as the neutralizer and the base–acid catalyst, respectively (30). In addition to these catalytic residues, His167 (neighboring histidine to the base–acid) and Tyr243 (=Tyr202 in FoRham1) are conserved between FoRham1 and LOR_107 (Fig. 9*B*). The catalytic residues of PLSV_3936 were suggested to be Arg204 (neutralizer), Tyr188 (base catalyst), and His123 (acid catalyst) (34). Structural superimposition at the catalytic area indicates that His143 adjacent to the catalytic residues is conserved in FoRham1 (His105) (Fig. 9*C*).

**Figure 10:**
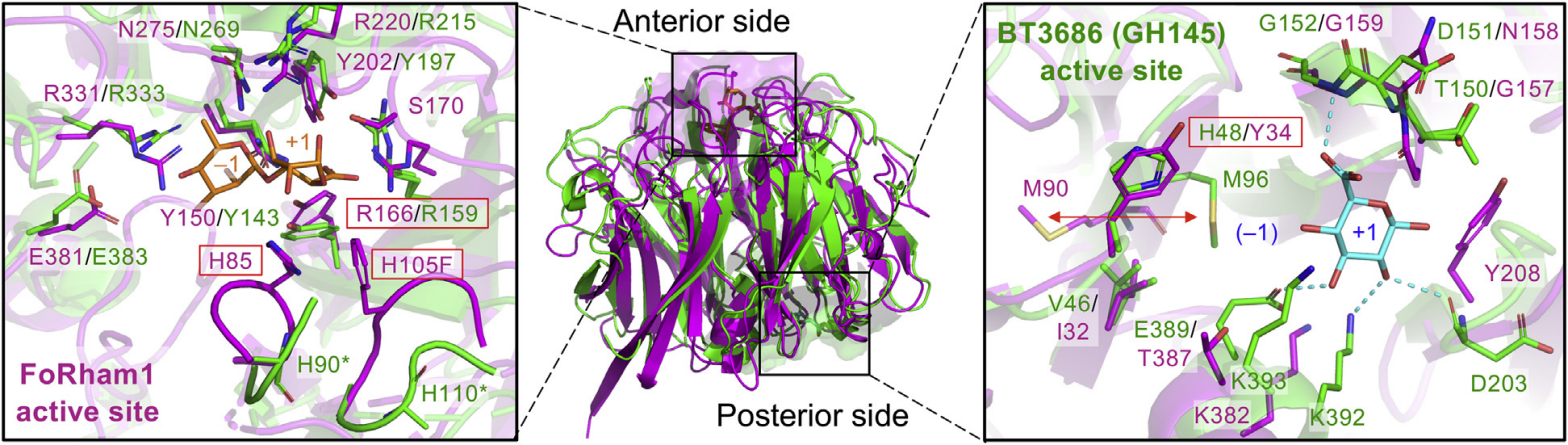
**Comparison with GH145.** Superimposition of the ribbon models of FoRham1 (*magenta*) and GH145 BT3686 (*green*, Protein Data Bank ID: 5MUL) is shown in the *middle*. *Left*, the active site of FoRham1 (*magenta*) on the anterior side superimposed with BT3686 (*green*). Rha–GlcA in FoRham1 is shown as *orange sticks*. The side chains of His90 and His110 in BT3686 (indicated by *asterisks*) are not modeled. *Right*, the active site of BT3686 (*green*) on the posterior side superimposed with FoRham1 (*magenta*). GlcA in GH145 BT3686 is shown as *cyan sticks*. Labels for the catalytic residues of FoRham1 and BT3686 are highlighted in *red boxes*.

Figure 10 shows a structural comparison with BT3686 (GH145), which is a retaining hydrolase cleaving the same glycosidic linkage (Rha–GlcA) with FoRham1. The active site of FoRham1 is located on the “anterior” surface that is usually exploited by most of the β-propeller enzymes, whereas the active site of BT3686 is on the opposite (posterior) surface (Fig. 10, *middle*). The posterior side of FoRham1 shows low amino acid conservation with the BT3686 active site (Fig. 10, *right*). His48 of BT3686 is the sole candidate of the catalytic nucleophile residue, but there is no candidate for the acid–base catalyst in the GH145 α-l-rhamnosidases (12). The catalytic histidine is replaced primarily by Gln (~25% of homolog sequences) and rarely by Asp, Phe, Tyr, or Ser. Munoz-Munoz *et al.* (12) could restore the activity of inactive GH145 members (BACCELL_00856, BACINT_00347, and BACPLE_00338) by Q to H or D to H mutation. The corresponding residue in FoRham1 is Tyr34. It is not clear if such mutation (Y34H) would create a retaining GH active on Rha–GlcA. However, the low amino acid sequence conservation at the posterior side implicates that this site is not involved in hydrolysis or substrate binding.

The left inset of Figure 10 shows a structural comparison of the FoRham1 active site with BT3686. Surprisingly, the active site residues of FoRham1, including the neutralizer (Arg166), are basically conserved in BT3686. In the crystal structures of BT3686, His90, and His110, which correspond to the base–acid catalyst (His85) and the neighboring histidine (His105) of FoRham1, are deviated from the active site, and their side chain was not modeled, probably because of disorder. The two histidine residues as well as the neutralizer arginine are highly conserved in GH145 members (Fig. S2). These facts strongly support the hypothesis raised by Munoz-Munoz *et al.* (12): the ancestral function of GH145 may be different from that of BT3686, and the retaining GH function at the posterior pocket of the *Bacteroides* enzymes is a recent innovation (neofunctionalization). Moreover, the resemblance of the anterior pocket of GH145 with PL25 ulvan lyase was already suggested. It is not clear why the *Bacteroides* enzymes did not show lyase activity, but the predominant function of the current GH145 members may be PL possessed by the fungal members (Fig. 2).

### Conclusion

We isolated and identified FoRham1 from the fungus, *F. oxysporum*, as a novel Rha-releasing enzyme specific to GA. Enzymatic characterizations indicated that FoRham1 was an l-rhamnose-α-1,4-d-glucuronic acid lyase, which was firstly found from a eukaryotic origin. The substrate recognition of FoRham1 was revealed in detail by a crystallographic study, and its catalytic mechanism was proposed according to the three-dimensional structure and the mutational analysis on the active site. The active site of FoRham1 is located on the usual anterior surface of the β-propeller fold. Because the lyase activity–related amino acid residues in the anterior pocket are conserved among the fungal homologs of FoRham1 and the *Bacteroides* members in GH145, the Carbohydrate-Active enZYme team will rename the former GH145 family into a novel PL family 42 (B. Henrissat and N. Terrapon, personal communication).

## Experimental procedures

### Substrates

*p*NP-α-l-Rha and GA (Lot number: 120M0035V) were purchased from Sigma–Aldrich Co. LWAG and rutin were purchased from Tokyo Chemical Industry Co, Ltd. RG-I oligosaccharides were prepared as described previously (37). GSOs (GSO4−7) were prepared from GA using the *Sphingomonas* exo-β-1,3-galactanase (Exo-1,3-Gal; (38)) as described previously (18). Rha–GlcA was prepared by incubating 0.1 mg of 4-*O*-α-l-rhamnosyl-β-d-glucuronidase (FoBGlcA; (18)) with 5% GA (500 ml) in 20 mM sodium acetate buffer (pH 4.0) at 37 °C for 48 h. The reaction product was recovered by ultrafiltration using a 10-kDa cut-off membrane (10K Minimate TFF Capsule with Omega membrane; Pall Life Sciences), concentrated by an evaporator, and purified by activated carbon resin (0–50% linear gradient of ethanol).

Ulvan was prepared from 10 g of dried *Ulva prolifera*, kindly provided by Mr Toshihiro Hotta (Kochi Prefectural Deep-Sea Water Laboratory), which was crushed in ethanol using a mixer and washed three times with 1 l ethanol at 4 °C for 48 h. The residue was stirred in 2 l water at room temperature for 24 h twice to extract polysaccharides. After centrifugation (15,000*g* for 20 min), the supernatant was concentrated using an evaporator and ultracentrifuged (130,000*g* for 50 min). The supernatant was then concentrated, dialyzed against water, and mixed with 2.5× ethanol, yielding water-soluble polysaccharides. The crude sample was dissolved in 0.1 M NaCl, applied to a HiLoad 16/600 Superdex 75 prep grade column (GE Healthcare UK), and eluted at a flow rate of 1 ml/min. Polysaccharides with a high molecular mass were used as the ulvan polysaccharide.

### Purification of native FoRham1 from *F. oxysporum* 12S culture

The culture supernatant (2.8 l) of *F. oxysporum* 12S, which was prepared as described previously (17), was concentrated by ultrafiltration (10K Minimate TFF Capsule with Omega membrane). The enzyme solution was loaded onto a Toyopearl DEAE-650M column (Tosoh Corp; 30 × 120 mm) equilibrated with 20 mM sodium acetate buffer (pH 5.0). Bound proteins were eluted by a NaCl linear gradient (0–1 M). Rha-releasing activity in each fraction was assayed using GA as the substrate. Active fractions were pooled, dialyzed against 20 mM sodium acetate buffer (pH 5.0), loaded onto a Mono Q HR 5/5 column (GE Healthcare), and eluted by a linear gradient of NaCl (0–0.5 M). Ammonium sulfate was added to the enzyme solution to 30% saturation, and the mixture was loaded onto a Resource phenyl column (6 ml) (GE Healthcare) equilibrated with 30% ammonium sulfate in 20 mM sodium acetate buffer (pH 5.0). The adsorbed proteins were eluted by a linear gradient of ammonium sulfate (30–0% of saturation). Finally, the active fractions were pooled, concentrated using a centrifugal cut-off filter (Nanosep 10K Ω; Pall Life Sciences), and loaded onto a Superdex 200 Increase 10/300 GL column (GE Healthcare) equilibrated with 500 mM NaCl in 20 mM Tris–HCl buffer (pH 8.0).

Protein content was determined using a Coomassie Plus Bradford Assay Kit (Pierce Biotechnology, Inc), with bovine serum albumin as the standard and with measurement of absorbance at 280 nm. Molecular masses of proteins were estimated by SDS-PAGE in 15% gels.

### LCMS–IT–TOF analysis

In-gel enzymatic digestion of FoRham1 after SDS-PAGE was performed using Trypsin Gold (Promega), and the digested peptide mixture was separated by a prominence nano-liquid chromatography system (Shimadzu). The sample was firstly loaded onto the trap column L-column2 ODS (0.3 × 5 mm, 5 μm) (Chemicals Evaluation and Research Institute) at a flow rate of 0.05 ml/min with mobile phase C (water, 0.1% formic acid) for 5 min, then the trapped samples were separated in a MonoSpray Monolithic C18 nano electrosprayer (50 μm × 50 mm; GL Sciences). The HPLC gradient was 5 to 70% mobile phase B (acetonitrile:water, 95:5, 0.1% formic acid) in mobile phase A (acetonitrile: water, 5: 95, 0.1% formic acid) at a flow rate of 2 μl/min for 90 min. Mass spectrometric analysis was performed in a data-dependent manner on a LCMS–IT–TOF (Shimadzu) equipped with a nano electrospray ionization source in positive-ion mode. The scan range was 50 to 1000 *m/z*. The voltage of electrospray ionization source and detector was 1.50 and 1.65 kV, respectively. The resulting data were submitted onto the Mascot search engine (http://www.matrixscience.com) linked with the NCBInr database to determine candidate peptides. Detailed Mascot search parameters are summarized in Table S3.

### Expression, purification, and mutagenesis

*P. pastoris* X-33 (Invitrogen) transformed with the pPICZαA (Invitrogen) vector containing the mature *Forham1* gene was obtained according to the previous report (39). FoRham1 WT, FoRham1 SeMet, and variants of FoRham1 were obtained using the same method as reported previously (18), except that 500 ml of a selenomethionine medium (40) was used for FoRham1 SeMet production. Site-directed mutagenesis to construct the variants was performed with the PrimeSTAR mutagenesis basal kit (Takara Bio) using the pPICZαA vector containing mature *Forham1* gene as the template and primers shown in Table S4.

The recombinant proteins expressed by *P. pastoris* were purified from the culture supernatants. The supernatant was concentrated with an ultrafiltration membrane (10K Centramate cassette with Omega membrane; Pall Life Sciences), dialyzed against 20 mM potassium phosphate buffer (pH 8.0), and loaded onto a Ni Sepharose 6 Fast Flow column (GE Healthcare, 25 × 50 mm). The column was washed with binding buffer (20 mM potassium phosphate buffer, 150 mM NaCl, and 20 mM imidazole; pH 8.0), and binding proteins were eluted with elution buffer (20 mM potassium phosphate buffer, 150 mM NaCl, 250 mM imidazole; pH 8.0). After dialysis against 20 mM Tris–HCl (pH 8.0), the enzyme was loaded onto a Mono Q GL 5/50 column (GE Healthcare) equilibrated with the 20 mM Tris–HCl (pH 8.0). Bound proteins were eluted by a NaCl linear gradient (0–0.5 M). The elution fractions were recovered based on the results of SDS-PAGE and native-PAGE. Rha-releasing activity in each fraction was assayed using GA as the substrate.

### Enzyme assays

A standard assay of FoRham1 was performed with GA as the substrate. The reaction mixture contained 200 μl of 0.1% GA in 50 mM Hepes–NaOH (pH 7.0) with the enzyme sample, which was incubated at 30 °C for 15 min. The mixture was then boiled for 5 min to inactivate the enzyme. After centrifugation, the Rha content of the supernatant was quantified by HPAEC-PAD as described later. One unit of enzyme activity was defined as the amount of enzyme required to release 1 μmol of Rha in 1 min under the aforementioned conditions.

Substrate specificity was measured using 0.1% GA, LWAG, ulvan, RG-I oligosaccharides, or 0.5 mM *p*NP-α-l-Rha, Rha–GlcA, and rutin as substrates in 50 mM Hepes–NaOH (pH 7.0). The reaction was performed at 30 °C for 30 min to 16 h. After boiling the mixture for 5 min, the released Rha was quantified by HPAEC-PAD. To assess the activity to ulvan, the increase in absorbance at 235 nm was measured after the enzymatic reaction.

Enzyme kinetics were determined by incubating the reaction mixtures containing the substrates (0.4–5%) in 50 mM Hepes–NaOH (pH 7.0) and FoRham1 (20 nM) at 30 °C for 30 min. The released Rha was quantitated by HPAEC-PAD. The *K*_m_ and *k*_cat_ values were calculated using the nonlinear regression plot at eight substrate concentrations. GA-RG used as the substrate is the reaction products of GA with FoBGlcA.

The enzyme kinetics of FoRham1 toward the oligosaccharide substrates were determined by incubating the reaction mixture comprising 20 nM enzyme and substrate (0.1–25 mM) in 50 mM Hepes–NaOH (pH 7.0) at 30 °C for 30 min. All kinetic parameters were calculated using the nonlinear regression plot at eight substrate concentrations.

The specific activity of FoRham1 toward oligosaccharides containing Rha–GlcA in a molecule was determined by incubating 1 mM substrate in 50 mM Hepes–NaOH (pH 7.0) and 0.2 μM FoRham1 at 30 °C for 5 min.

The relative activities of FoRham1 WT and the mutant enzymes were analyzed using GA (1%) and Rha–GlcA (1 mM) as the substrate. The enzymes (5 μM) were incubated with each substrate in 50 mM Hepes–NaOH (pH 7.0) at 30 °C for 5 min.

### Yield of Rha released from GA with FoRham1

A reaction mixture containing 1% GA (10 ml) in 50 mM Hepes–NaOH (pH 7.0) and 0.1 mg FoRham1 was incubated at 30 °C for 48 h and boiled to inactivate the enzyme. The mixture was then dialyzed against water to remove Rha and hydrolyzed in 1 M sulfuric acid at 100 °C for 1 h. After neutralization by NaOH solution, the Rha content in the hydrolysate was measured using HPAEC-PAD, and the content before and after the enzyme reaction was compared.

### HPAEC-PAD condition

HPAEC-PAD was performed using a Dionex ICS-5000 system (Thermo Fisher Scientific) with a CarboPac PA-1 column (4 × 250 mm; Thermo Fisher Scientific). Carbohydrate samples were eluted at a flow rate of 1 ml/min with 0.1 M NaOH for 5 min followed by a linear gradient from 0 to 0.45 M sodium acetate in 0.1 M NaOH for 30 min, and eluates were monitored with PAD.

### NMR spectroscopy experiment

The reaction mixture containing FoRham1 (40 nM) and 1% GA (5 ml) in 20 mM MES–NaOH (pH 7.0) was incubated at 30 °C for 48 h and boiled for 5 min to inactivate the enzyme. After centrifugation and filtration using 0.45 μm membrane, the solution was dialyzed against water for 24 h at room temperature to remove reaction products and lyophilized. To substitute ^1^H with D, a purified carbohydrate sample (10 mg) was dissolved in 0.3 ml heavy water (D_2_O), lyophilized, and dissolved in 0.6 ml D_2_O. The ^1^H-NMR spectra were recorded with a 500-MHz NMR spectrometer (JNM-ECZR500; JEOL Ltd) in D_2_O at 60 °C and analyzed with Delta NMR Processing and Control version 5.2.1 software (JEOL Ltd). Acetone (δ_H_ = 2.225 ppm) was the internal standard and accumulated 32 times.

### Crystallography and structure determination

Crystallization experiments were performed using the sitting-drop (Rha complex, N247A, and H105F) or hanging-drop (WT and SeMet) vapor diffusion method. Two microliters of protein solution was mixed with an equal volume of crystallization solution and vapor diffused to obtain 500 μl of the crystallization solution with 24-well crystallization plate. The initial protein concentration of FoRham1 WT used for crystallization was determined using a PCT kit (Hampton Research). Crystal screen HT and PEG Rx HT (Hampton Research) with a 96-well crystallization plate (ASONE) were used for crystallization screening of FoRham1 WT (10 mM Tris–HCl, pH 8.0) concentrated to 24 mg/ml. The crystals of FoRham1 WT were obtained by mixing the protein solution with the reservoir solution (PEG Rx HT F6) for approximately 5 days at 20 °C. Screening was performed with a drop ratio of 1:1. The Rha-complexed crystal was prepared by cocrystallization with 100 mM Rha. FoRham1 SeMet (17 mg/ml in 10 mM Tris–HCl, pH 8.0) crystals and N247A (24 mg/ml in 10 mM Tris–HCl, pH 8.0) crystals were prepared in the same reservoir solution as that used for FoRham1 WT crystals for approximately 5 days at 4 °C. Crystals of H105F (27 mg/ml in 10 mM Tris–HCl, pH 8.0) were obtained by mixing the protein solution with 0.1 M sodium acetate (pH 4.6) and 1.6 M ammonium sulfate for approximately 1 week at 4 °C. For Rha–GlcA complex crystals, crystals of H105F mutant were soaked in the reservoir solution containing 20 mM Rha–GlcA and 20% glycerol at 25 °C for <1 min. Crystals in 24-well plates, except for H105F, could not be obtained with homemade crystallization solutions, so mixed solutions purchased from Hampton Research were used. All crystals except for those of H105F were soaked for <1 min at 25 °C in a crystallization solution containing 20% glycerol as a cryoprotectant. All the prepared crystals were flash cooled by soaking in liquid nitrogen.

X-ray diffraction data were measured at the beam line of the Photon Factory and Photon Factory Advanced-Ring of the High Energy Accelerator Research Organization KEK. X-ray diffraction images of Rha-complex crystals were collected with the AR-NW12A beamline (detector; PILATUS3 S2M). X-ray diffraction images of the other crystals were collected with the BL-5A beamline (detector; PILATUS3 S6M).

The diffraction images were processed with XDS (41), and the statistics were calculated with AIMLESS (42). The initial phase and automatic model constructions were calculated with PHENIX (43). The ligand complex structures were determined by the molecular replacement method with Molrep (44) using the ligand-free structure as a search model. Manual model construction and refinement were performed with Coot (45) and REFMAC5 (46). The statistics for data collection and refinement are listed in Table 5. Molecular graphic images were prepared with PyMOL (Schroedinger LLC).

## Data availability

Atomic coordinates and structure factors (codes 7ESK, 7ESM, 7ESN, and 7ESL) have been deposited in the Protein Data Bank (http://wwpdb.org/).

The MS proteomics data have been deposited to the Figshare repository (https://figshare.com/) with the dataset identifier 10.6084/m9.figshare.14814033 for raw mass spectrometry data, 10.6084/m9.figshare.14634492 for the Mascot search results, and 10.6084/m9.figshare.14634651 for the mgf file.

## Supporting information

This article contains supporting information (12).

## Conflict of interest

The authors declare that they have no conflicts of interest with the contents of this article.
