# Supplementary material for: Structural and functional analysis of gum arabic l-rhamnose-α-1,4-d-glucuronate lyase establishes a novel polysaccharide lyase family
Source: J Biol Chem. 2021 Jul 23;297(3):101001. doi: 10.1016/j.jbc.2021.101001 (PMC8377490; doi:10.1016/j.jbc.2021.101001)
Supplement: Supplemental Figures S1–S9 and Tables S1–S4 [file mmc1.pdf]

Supporting information

Structural and functional analysis of gum arabic L-rhamnose-α-1,4-D-glucuronate lyase establishes a novel polysaccharide lyase family

Tatsuya Kondo[1], Miyu Kichijo[1], Akiho Maruta[1], Makoto Nakaya[2, 3], Shigeo Takenaka[4], Takatoshi Arakawa[5, 6], Shinya Fushinobu[5, 6], and Tatsuji Sakamoto[1,*]

[1]Graduate School of Life and Environmental Sciences, Osaka Prefecture University, Sakai, Osaka 599-8531, Japan. [2]Center for Research and Development of Bioresources, Organization for Research Promotion, Osaka Prefecture University, Sakai, Osaka 599-8570, Japan. [3]Department of Nutrition, Otemae College of Nutrition and Confectionery, Osaka, Osaka 540-0008, Japan. [4]Graduate School of Comprehensive Rehabilitation, Osaka Prefecture University, Habikino, Osaka 583-8555, Japan. [5]Department of Biotechnology, The University of Tokyo, Tokyo 113-8657, Japan. [6]Collaborative Research Institute for Innovative Microbiology, The University of Tokyo, Tokyo 113-8657, Japan.

[*] Corresponding author: sakamoto@biochem.osakafu-u.ac.jp

Figure S1. SDS- and native-PAGE analyses of native and recombinant FoRham1.

Figure S2. Multiple sequence alignment prepared with Clustal Omega and ESPript 3.0.

Figure S3. Effects of temperature and pH on recombinant FoRham1 activity and stability.

Figure S4. Kinetic analysis of FoRham1 using non-linear regression fitting.

Figure S5. C-terminal swapping of H105F Rha-GlcA complex structure.

Figure S6. N-glycosylation.

Figure S7. Effect of metal ions on the enzyme activity of FoRham1.

Figure S8. Ca^{2+} ion binding site of FoRham1 WT.

Figure S9. Superimposition of FoRham1 with structural homologs.

Table S1. Information on peptides identified based on MS/MS spectral data.

Table S2. Result of structural similarity search with the Dali server using the FoRham1 WT structure.

Table S3. The Mascot search parameters.

Table S4. Sequences of primers for mutagenesis.

Supplementary Figure 1

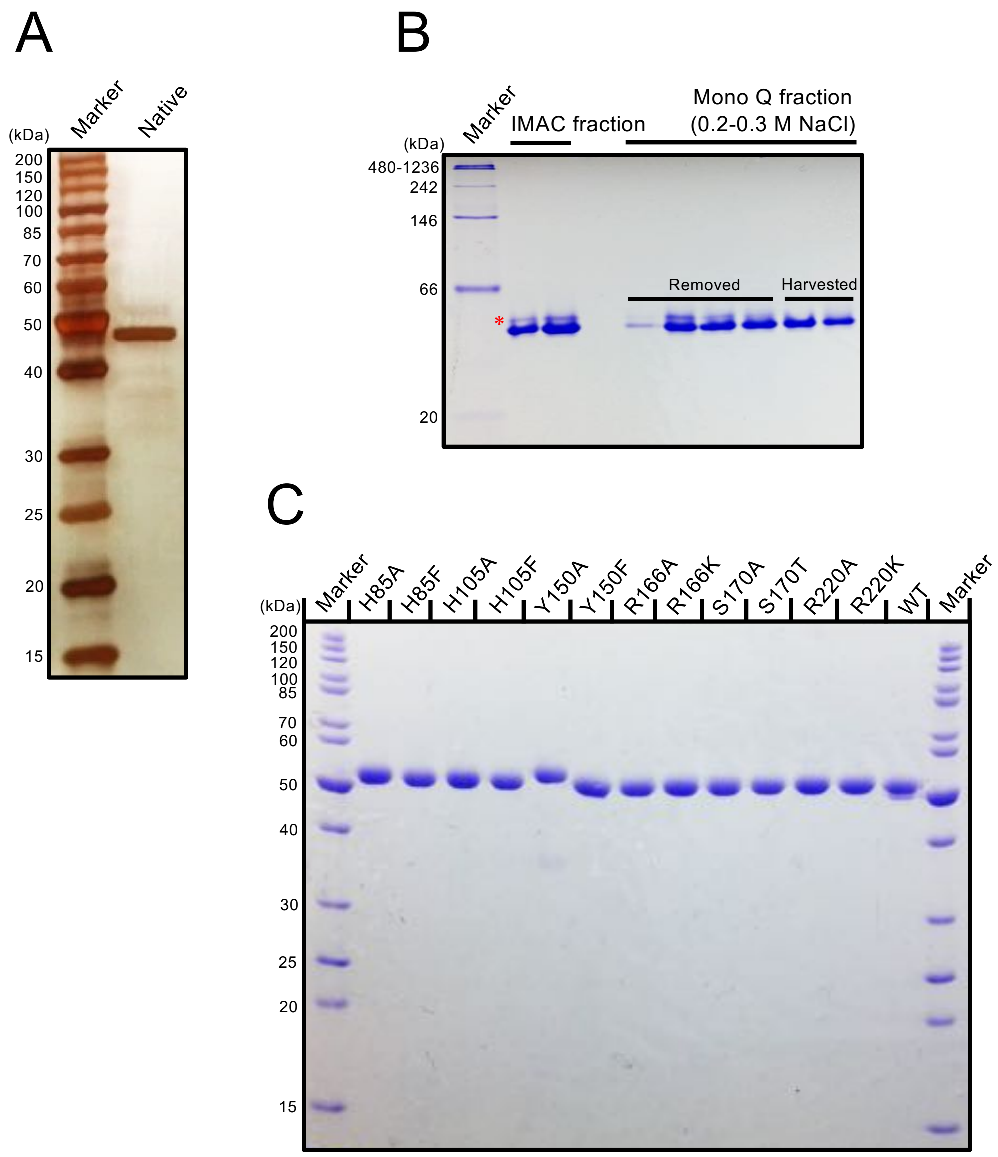

Figure S1. SDS- and native-PAGE analyses of native and recombinant FoRham1. *A*, Purified native FoRham1 was analyzed by SDS-PAGE and visualized by silver staining. *B*, Recombinant FoRham1 after IMAC and anion-exchange chromatography was visualized by Native-PAGE (12% polyacrylamide gel) and CBB R-250. The asterisks in the picture indicate the contamination bands. *C*, Purified recombinant FoRham1 (WT and mutants) was analyzed by SDS-PAGE and visualized by CBB R-250 staining.

Supplementary Figure 2

```
                                                        1        10        20
FoRham1            ......................................................LTVKSTKQWTIGTDVQGSERLNGVSYQE.............
FFUJ_14848         ...............................MSAVSA...................ITVKSTKQWTIGTDVQGSQRLNGVSYQD.............
FGRAMPH1_01T21617  ..............MRLSSIFLSLTSLMATTTA..............LKIKSTKQWTIGTDIQGSERLNGVSYQE.............
FVRRES_10681       ..............MRLSSIIFSLASLMATTTA..............LTVKSTKQWTIGTDIQGSERLNGVSYQE.............
FVRRES_08057       ..............MANLKSLIYLAALSAVHAT..................VTPGSLTTISTAVDANSQAA..............
LOR_107            MKINLSMRELVSRLSTTLKTAIALSVLTACTANDSVSLTSNISNTSGVLLESQTKITDGALHFDGKKLNHNTFENPSKSQAYDYFFGR
LOR_61             .............MKCLKTLLVSTTLLTAFSLN...........AEVTLEQQVKITEEGLHFDGRNLDFSNVGTPDTGEKYDYFFGP
PLSV_3875          MKLNLKASGVARQLTTLAKTVAALSVLTACASSNTATVSSAMSKANGVFLESQTKITNGALHFDGKKLNHNTFEKPSLGPEYDYFFGK
PLSV_3925          ..............MNGLKMLLFSTTLLTAFTLH...........AQVTLKQQVKITDEGLHFDGRNLDFSNVGTPDTGEKYDFFFGP
BACCELL_00856      ...............MNRYILFFFLILSTLTT..............LRAQQSRLVEVGNGYSQTS.VNTTVFRN...........
BT3686             ...............MKRTLLLTFLLLS.VSA..............LCFSQ.KLVEVAKGYSCTS.VNTTIFRN...........
HMPREF9455_02360   ..............MKIKKYLLISIILFS....................LSAHAQKLIPVAPGWSNNS.VNVTVFRK...........
```

```
                                                                                   H85                H105
                   30        40        50        60        70        80        90        100
FoRham1            ......DALITYGDYQYVTFYETAPAG..YLNHFVKVGRRRVSPSVGDWEFLTLDDYTQK..TMDGHNMISMGISG.DGKIHLSFDHH
FFUJ_14848         ......DALITYGDYQYVTFYETAPAG..YLNHFVKVGRRKLSPSVGDWEFLSLDDYIQK..TMDGHNMISMGISG.DGKIHLSFDHH
FGRAMPH1_01T21617  ......DALVTYGNYQYVTFYETAPAG..YLNHFVRLGRRKISPSIADWEYLTLDDYTQK..TMDGHNMISMGISG.DGKIHLSFDHH
FVRRES_10681       ......DALITYGDYQYVTFYETAPAG..YLNHFVRLGRRKISPSVADWEYLTLDDYTQK..TMDGHNMISMGISG.DGKIHLSFDHH
FVRRES_08057       ....WWNPLDEYGGYDWLAYLQNPPGGSTANNNVMVARRSISDGIISRDCLKTSAGDCAVFADDLGHNTPSISVDG.DGYVHVFTSMH
LOR_107            NISAHGDAVKPYKHFVFMTWYKGGKEE..R.NVMLSRFNTKTGVVKTIQFPHRHTGFRGDPLVGESHNTIGLAVSPLNGTIHMVYDMH
LOR_61             NISAHGDAVKTYRHYVFMTWYKGGKNE..R.NVMLSRYNTLNGELSTIEFPHKHTGFRGDPLVGESHNTIGLSVSPINGTIHMVFDMH
PLSV_3875          NISAHGDAVKPYKHYVFMTWYKGGKEQ..R.NVMLSRFNTKTGVVKTIQFPHRHTGFRGNPLVGESHNTIGLAVSPKNGTIHMVYDMH
PLSV_3925          NISAHGDAVKTYKHYVFMTWYKGGKSE..R.NVMLSRYNTLSGELSTIEFPHRHTGFRGDPLVGESHNTIGLSVSPINGTIHMVFDMH
BACCELL_00856      ......NSLVTQGDEQYISYYDGD........GYLVLGKRKLD...SDQWTLKRTQYKGN..VKDAHNIISMMLDG.EGYLHLSFDHH
BT3686             ......NSLVTHGDEQYISYYDAD........GYLVLGKRKLN...SKQWTLHRTQYRGN..VKDAHNIISIMVDG.EGYLHVSFDHH
HMPREF9455_02360   ......NSLVTHNNVQFIAYYDPD.........GFLTLGKRNIG...EDSWTISKTQYKGN..VSDAHNSISIMTDG.DGYLHVSWDHH
```

```
                                                                                     Y150
                                 110        120        130        140                 150       160
FoRham1            DVP................INYRISKNGIAKDVPSKWTSDLFDPVVHELVGSQ...............GPYSPLTYPRFEPLGNGDLL
FFUJ_14848         DVP................INYCISKNGIAKDVPSEWTSNLFGPVVHELVGSQ...............GPYSPLTYPRFQPLSNGDLL
FGRAMPH1_01T21617  DVP................INYRVSKAGIAKDVPSKWSSELFGPVVHTLDGSQ...............GPYSPLTYPRFEPLPNGDLL
FVRRES_10681       DVP................INYRVSKAGIAKNIPSKWSSELFGPVIHSLDGST...............GPYSPLTYPRFEPLPNGDML
FVRRES_08057       NEP................WQYFRSATPYSSTLVDAS.........SEMPDQT...................TKFTYPVIKRDADGNLW
LOR_107            AYVDDDETGRFKGRFVDDFFRYSFSVAGAADVPDDEFTLEQFVKDTSELSQGADDYKHLTMTGNLQDKENFSALTYPKFYTSDDGELL
LOR_61             AYDNNNHGGKFK....DDFFRYSFSVAGAAELPHSEFTLDKFVKDTSEVSQGDDDYKHLTMTGDLDDKGNFARLTYPKFFTTVDGTLL
PLSV_3875          AYVDDDESGRFKGRFVDDFFRYSFSVPGAADVPDDEFTLEKFVKDTSEVSQGTDDFKHLTMTGNLEDKDNFSALTYPKFYKSKEGELL
PLSV_3925          AYDNNNHDGKFK....DDFFRYSYSIAGAAELPHSEFTLDKFVKDTSEVSQGENDYKHLTMTGDLSDKGNFARLTYPKFFTTVDGTLL
BACCELL_00856      GHK...............LNYCRSTAPYTLELGDKE......PMTGVDEGN...................VTYPEFYPLNGGDLL
BT3686             GHK...............LNYCRSIAPGSLELGDKM......PMTGVDEGN...................VTYPEFYPLTDGDLL
HMPREF9455_02360   GHP...............LRYAKGIAPYSLELGDKQ......PMTGSLETN...................VTYPEFLKMPDGDLI
```

```
                      R166                                                        Y220
                            170        180        190        200        210        220        230
FoRham1            LEFR...IGQSGSGDSYIHRYSASTGKWQ.AYGMYIQ.....GDDN.NAYINGLDYLDGKLYTSWTVRETPNADTN...HGVYFAYSN
FFUJ_14848         IEFR...IGQSGSGDSYIHRYSASTGKWQ.AYGMYIQ.....GDDN.NAYINGLDYLDGKLYTSWTVRETPNADTN...HGVYFAYSN
FGRAMPH1_01T21617  LEFR...IGQSGSGDSYIHRYSSSTGKWQ.AQGMYIQ.....GDDN.NAYINGLDYLDGKLYTSWTVRETPNADTN...HGVYFAYSN
FVRRES_10681       LEFR...IGQSGSGDSYIHRYSSSTGKWQ.AQGMYIQ.....GDDN.NAYINGLDYLDGKLYTSWTVRETPNADTN...HGVYFAYSN
FVRRES_08057       LIIRGQAPNDNNAKGGYFYKYSTDDGSWSRVAIWAYN......KGYSVYPDDIAFSSDGDVHLQWEWSKYPASAVR...HQGSYVRYN
LOR_107            HYMR...WGGNNNGAYYFNKYDAKNQKWTRFTPFNHKDQKTHGNAYNWGLYGQMKYINGKLRVGFQQRSANNDDRFKYQNGVYYAYSD
LOR_61             LYMR...LGGNNNGAYVFNRYDAETESWSTFTKFNENNQKLKGNQYNWGLYGNMKYVNGKLRVGFQQRSNDNSDKYKYQNGVYYAYSD
PLSV_3875          HYMR...WGGNNNGAYYFNKYDAEKQVWTRFTPFNHKDQETHGNAYNWGLYGQMKYINGKLRVGFQQRSANNNDRYKYQNGVYYAYSD
PLSV_3925          LYMR...LGGNNNGAYVFNRYDAETETWSTFTKFNENNQKLKGNPYNWGLYGNMKYVNGKLRVGFQQRSNDNSDKYKYQNGVYYAYSD
BACCELL_00856      FVYR...SGSSGRGNLVMNHYSVKEKKWNRVQDVLID.....GEDQRNAYWQLYVDEQGTIHLSWVWRETWHVETN...HDLCYARSY
BT3686             FVYR...SGSSGRGNLVMNRYSLKDHKWARVQDVLID.....GEDKRNAYWQLYVDEKGTIHLSWVWRETWQVETN...HDLCYARSF
HMPREF9455_02360   FMYR...DGQSGRGNLVMNRYNQKSKTWTQIQKNFID.....GENQRNAYWQSCIDDKGIIHLSWVWRETADVATN...HDLCYARSS
```

```
                   240        250        260        270        280        290        300        310
FoRham1            DDG..KTWFNTNDTKLTKPISTSDDSTLIWD.......IPQNSRMVNQEGQLIDTKGRFHILMRDLLSGEHQYQHYLRKADG......
FFUJ_14848         DDG..KTWFNTNGTKLSKPISTSDDSTLIWD.......VPQNSRMVNQEGQLIDTKGRFHILMRDMLSGEHQYQHYLRKTDG......
FGRAMPH1_01T21617  DDG..MTWFNTNGTKLTKPISTSDASTLIWD.......IPQNSRMVNQEGQLVDTKGRFHILMRDLLSGKHLYQHYLRDTNG......
FVRRES_10681       DDG..KTWLNTNGTKLTKPFSTSDASTLIWD.......IPQNSRMVNQEGQLVDTRGRFHILMRDLLSGEHLYQHYLRDTNG......
FVRRES_08057       PST..KAFTSASGDTVSVPVTQNSADVVYQP.......LTSGETYSGDINASPGPAFQSAKMALYERDGSLHIQHAYRFKTSN.....
LOR_107            HPDGLGNWKNVDGEDMTWPLVNSDEIKIFEPGDYIDHTAPNSVHIVTGFDWTVTENDDVHFITHVRSTDTKRSDYKEVSIHA......
LOR_61             HPDGFGDWKNHKGEPMTWPLINSDEIKVFEPGDYISHTEANSVYIVGSFDWTVTEKGDIHIISKVRSTDRGRADYEEVYIHS......
PLSV_3875          HPDGLGDWKNVDGENMTWPLVDSDEIKIFEPGDYIDHQEPNSVHIVGGFDWTVTENEDLHFITHVRSTNTKRSDYKEVSIHA......
PLSV_3925          HPDGFGDWKNHKGEPMTWPLINSDEIKVFEPGDYVSHTDANSVYIVGSFDWTVTEKGDIHIISKVRSTDRSRPDYEEVYIHS......
BACCELL_00856      DNG..VTWYKANGKKYDLPIRYNN.AEYACR.......IPQNSELINQTSMSADASGNPYIATYWRDPDSEVPQYRIVWHDGQMWHNR
BT3686             DNG..VTWYKSDGEQYKLPITASN.AEYACR.......IPQNSELINQTSMSADAGGNPYIATYWRSSDSEVPQYRIVWNDGKTWHNR
HMPREF9455_02360   DGG..VSWENSKGEKYSLPITVAK.SEIACP.......IPQNSELINQTSMVTDAKGNPYIATYWREQDSDIPQYHIVYYTGKKWNDL
```

Continued to next page

Supplementary Figure 2

```
                                   320       330       340       350       360       370         380       390
FoRham1              ....TWTKNAINPAGLNGPDLYDPRGKLAGDASGEYLFGILPDPVKQSTGIYVATASKDFKDWKS..LAEIPNTSTEPLFDKTRLHES
FFUJ_14848           ....TWTKNAISPAGLNGPDLYDPRGKLAGDASGEYLFGILPDPVKQSTGIYVATASKDFKDWKS..LAEIPNTSTEPLFDKTRLHES
FGRAMPH1_01T21617    ....KWTKNAINPAGLNGPDLYDPRGKLAGDATGEYLFGLLPDPVKQSTGIYVATASKGFKDWMS..LAEIPNTATEPLFDRTRLHEF
FVRRES_10681         ....KWTKNAINPAGLNGPDLYDPRGKFAGDASGEYLFGLLPDPVKQATGIYVATASKGFKDWAS..LAEIPNTSTEPLFDKARLHEF
FVRRES_08057         .AGPWQVRRATGTYGTQDPWKREILYQDSETSATVGITHDGTTARIYYCRSSASAYVLEKFGGAGWTNTALEPTVGKKVQRLQAVMRS
LOR_107              ....FKPANAVDFTITTDFTGADSIYTSGDSIFIIGLKNGYPFVEKAKGGSNDFEVVYQQASGVKFDHGTIHIENGKAYYYLMEKGAG
LOR_61               ....YKPAGAEEFIISTDFPGASEIYTSGDNVYIVGLEGGRPYVEKAQGGTNNFIRVYEASDGPVFDHGTLYIKDGKVYYYLMERTSG
PLSV_3875            ....FKPANAKDFTVTTDFTGADSIYTSGDSIFIIGLKNGYPFVEKAKGGTNEFEVVYQQTSGVKFDHGTIHIENGKAYYYLMEKGAG
PLSV_3925            ....YKPAGAEDFIISTDFTGASEIYTSGDNVYIVGLEGGRPYVEKAQGGTNNFVRVYEASDGPTFDHGTLYIKDGKVYYYLMERTSG
BACCELL_00856        QVSNRHTPFSLKGGGTKMIPIARPRIVVDGG....EIFYIFRDEERGSRVSLAHAAAVGVGKWTFTDLTNFPVDAWEPSHDTELWKQK
BT3686               QVTDRKTPFTLKGGGTKMIPVARPRIVVEDG....EIFYIFRDEERGSRVSMAHTADVANGKWIVTDLTDFSVDAWEPSHDTELWKKQ
HMPREF9455_02360     NLGFRKTPFSLKGMGTKRIPISRPQVVAKVKGKSTELYLLFRDEERGEKASVAICKDIKKNKWEISDLTDVSLGSWEPSFDTELWREK

                       400       410
FoRham1              GILSVFVRQAG.GFPDRKLQVWDFEFDL............................................................
FFUJ_14848           GILSVFVRQAG.GFPDRKLQVWDFELDL............................................................
FGRAMPH1_01T21617    GVLSVFVRQAG.GFPDRKLQVWDFELDL............................................................
FVRRES_10681         GVLSVFVRQAG.GFPDRKLQVWDFELDL............................................................
FVRRES_08057         DGTDVLYLGSPTNVNSTTGSLYLVTVGGRD..........................................................
LOR_107              NALPLHLQVIDLGVTE........................................................................
LOR_61               TAMPLYLQIIDLDL..ESDANAPIVSFPSPSVTVNQGYEKLSLNISAESPVEGRSIQSVSLYIDDELVRTDDSLPFLFGHGSKPHETG
PLSV_3875            NSLPLHLQVIDLGVSR........................................................................
PLSV_3925            NAMPLYLQIIDLDLDLESDANAPIVSFPSPSLTVEQGFEKLSLNIAAESPVEVRTIQSVTLYINDELVRTDTSLPYLFGHGSKPHETG
BACCELL_00856        RRLHLFVQHTKQGDGERRVEFAPQPVYYVLEVVKPKGD..................................................
BT3686               RKLNLFVQHTCQGDGERTAEIEPQMIYVLEANTNTKK...................................................
HMPREF9455_02360     GLLHVFIQKVEQVDGEGKADVEPQMVNVLEVE........................................................

FoRham1              ........................................................................................
FFUJ_14848           ........................................................................................
FGRAMPH1_01T21617    ........................................................................................
FVRRES_10681         ........................................................................................
FVRRES_08057         ........................................................................................
LOR_107              ........................................................................................
LOR_61               ALGWLDRHEPNPSPLSAGRHVFKAVAVDSEGDSSTATMILNVNSNAPIVSFPQESLEVDEGFERLSLNISAESAVEGRTIESVSLYID
PLSV_3875            ........................................................................................
PLSV_3925            AMGWLDTHEPNPSPLPAGRHIFKAVAVDSEGDSSVATMMLTVNSNAPIISFPQESLEVDEGFEKLSLNISAESAVEGRTIESVSLYID
BACCELL_00856        ........................................................................................
BT3686               ........................................................................................
HMPREF9455_02360     ........................................................................................

FoRham1              ........................................................................................
FFUJ_14848           ........................................................................................
FGRAMPH1_01T21617    ........................................................................................
FVRRES_10681         ........................................................................................
FVRRES_08057         ........................................................................................
LOR_107              ........................................................................................
LOR_61               GGLVRTDTSLPYLFGHASKPHETGAMGWLDTHSPNPSPLAAGSYEFTAVATDNEGEETTASMLLVVKGEPEPPIVTWPNSTVTVYEGY
PLSV_3875            ........................................................................................
PLSV_3925            GEFVRTDTSLPYLFGHASKPHETGAMGWLDTHSPNPSPLTSGTYEFTAVAIDSEGEESTATMQLVVKGEPEPPVVTWPNSTVTVYEGY
BACCELL_00856        ........................................................................................
BT3686               ........................................................................................
HMPREF9455_02360     ........................................................................................

FoRham1              ........................................................................................
FFUJ_14848           ........................................................................................
FGRAMPH1_01T21617    ........................................................................................
FVRRES_10681         ........................................................................................
FVRRES_08057         ........................................................................................
LOR_107              ........................................................................................
LOR_61               EKLAITIDAETPVEGRDIQSVTLFRNGELVRVDTRPVWNFGHSFAPYEFGAMGWLDRHEPNPSPLGVGTHTFTAVARDSAGLESETDM
PLSV_3875            ........................................................................................
PLSV_3925            EKLAITIDAESPVEGRDIQSVTLYRNGELVRVDTRPVWNFGHSHAPYEFGAMGWLDRHDPNPAPLSVGTHTFTAVARDSAGLETESDM
BACCELL_00856        ........................................................................................
BT3686               ........................................................................................
HMPREF9455_02360     ........................................................................................
```

Continued to next page

Supplementary Figure 2

```
FoRham1             ........................................................................................
FFUJ_14848          ........................................................................................
FGRAMPH1_01T21617   ........................................................................................
FVRRES_10681        ........................................................................................
FVRRES_08057        ........................................................................................
LOR_107             ........................................................................................
LOR_61              ALIVLSLPGPSVMINEGDISLLTEYQNLAITAEASAADDDISLVSLALYIDEQLIREIYEPPFIWGSDAYSTELLSLTEGTHLVRVVA
PLSV_3875           ........................................................................................
PLSV_3925           TLIVLSLPGPSVMINESDISLLTEYQNLSITADASTANDDTSLVSLALYIDDQLVREIYEPPFEWGADGYSNELLELSEGSHLARVVA
BACCELL_00856       ........................................................................................
BT3686              ........................................................................................
HMPREF9455_02360    ........................................................................................
```

```
FoRham1             ..............................................................................
FFUJ_14848          ..............................................................................
FGRAMPH1_01T21617   ..............................................................................
FVRRES_10681        ..............................................................................
FVRRES_08057        ..............................................................................
LOR_107             ..............................................................................
LOR_61              TDSNNKQSESSIFINIDLLGDLNKDSIVDKADTRLFTSKLRAGEIMDIRYDFNGDGVVNNRDTRGLVRRCTYSRCSSN
PLSV_3875           ..............................................................................
PLSV_3925           TDSNNKQSESSIFINIDLLGDLNKDSVVDKGDTRLFTAKLRAGETMDIRYDFNGDGVVNNRDTRGLIRRCTYSRCTSN
BACCELL_00856       ..............................................................................
BT3686              ..............................................................................
HMPREF9455_02360    ..............................................................................
```

Figure S2. Multiple sequence alignment prepared with Clustal Omega and ESPript 3.0. Completely conserved amino acids are highlighted in red, and partially conserved residues are blue boxed. The GenBank accession numbers of the protein used for the alignment are as follows: FoRham1 (LC617219); FFUJ_14848 (CCT67810.1); FGRAMPH_01T21617 (SCB65023.1); FVRRES_10681 (CEI70604.1); FVRRES_08057 (CEI67980.1); LOR_107 (AMA19991.1); LOR_61 (WP_032096165.1); PLSV_3875 (AMA19992.1); PLSV_3925 (WP_033186955.1); BACCELL_00856 (EEF91490.1); BT3686 (AAO78791.1); HMPREF9455_02360 (EGK01332.1). The colored triangles indicate the catalytic residues (neutralizer and base/acid for green and cyan, respectively) and other important residues (red and grey) for the activity of FoRham1 (see Fig. 8 and Table 6). The partially conserved catalytic residue of GH145 (His48 of BT3686) is indicated by a white triangle.

Supplementary Figure 3

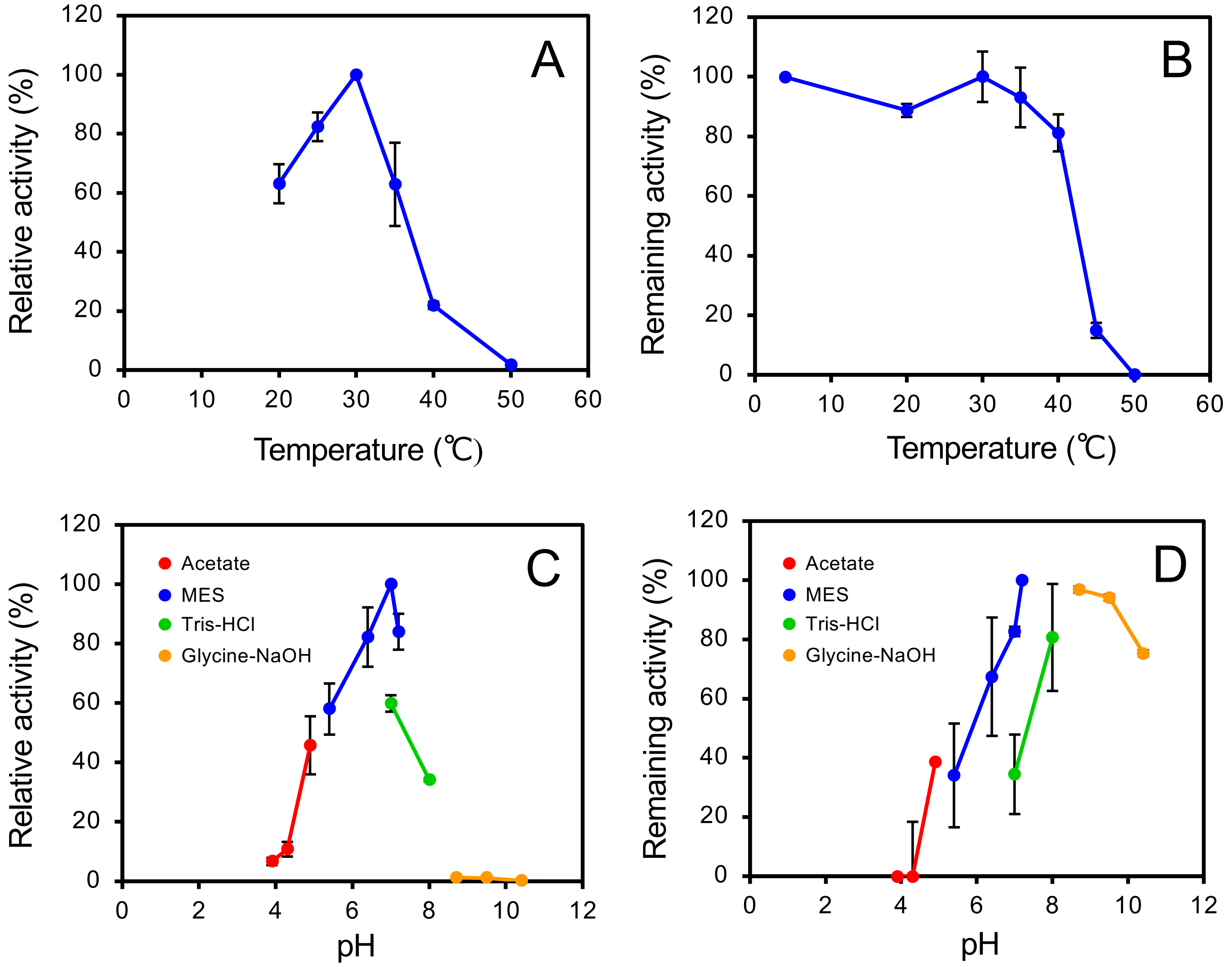

Figure S3. Effects of temperature and pH on recombinant FoRham1 activity and stability. *A*, Effects of temperature on the activity. Enzymatic reactions proceeded for 30 min at various temperatures in 20 mM acetate buffer (pH 5.0) with 0.1% (w/v) GA as the substrate. *B*, Effects of temperature on the stability. Enzyme stability was assessed by measuring residual enzyme activity after pre-incubation for 1 h at various temperatures in 20 mM acetate buffer (pH 5.0) with 100 µg/ml bovine serum albumin (BSA). *C*, Effects of pH on the activity. The enzymatic reactions with 0.1% (w/v) GA were run for 1 h in various buffers (50 mM; pH 3.9–10.4) at 37 °C. The buffers were acetate buffer (red), MES buffer (blue), Tris-HCl buffer (green), and glycine-NaOH buffer (orange). *D*, Effects of pH on the stability. Enzyme pH stability was assessed by pre-incubation at 37 °C for 1 h in the various buffers indicated in (C) above. The enzyme units used for all measurements were 0.2 mU. All values are reported as means ± SE.

Supplementary Figure 4

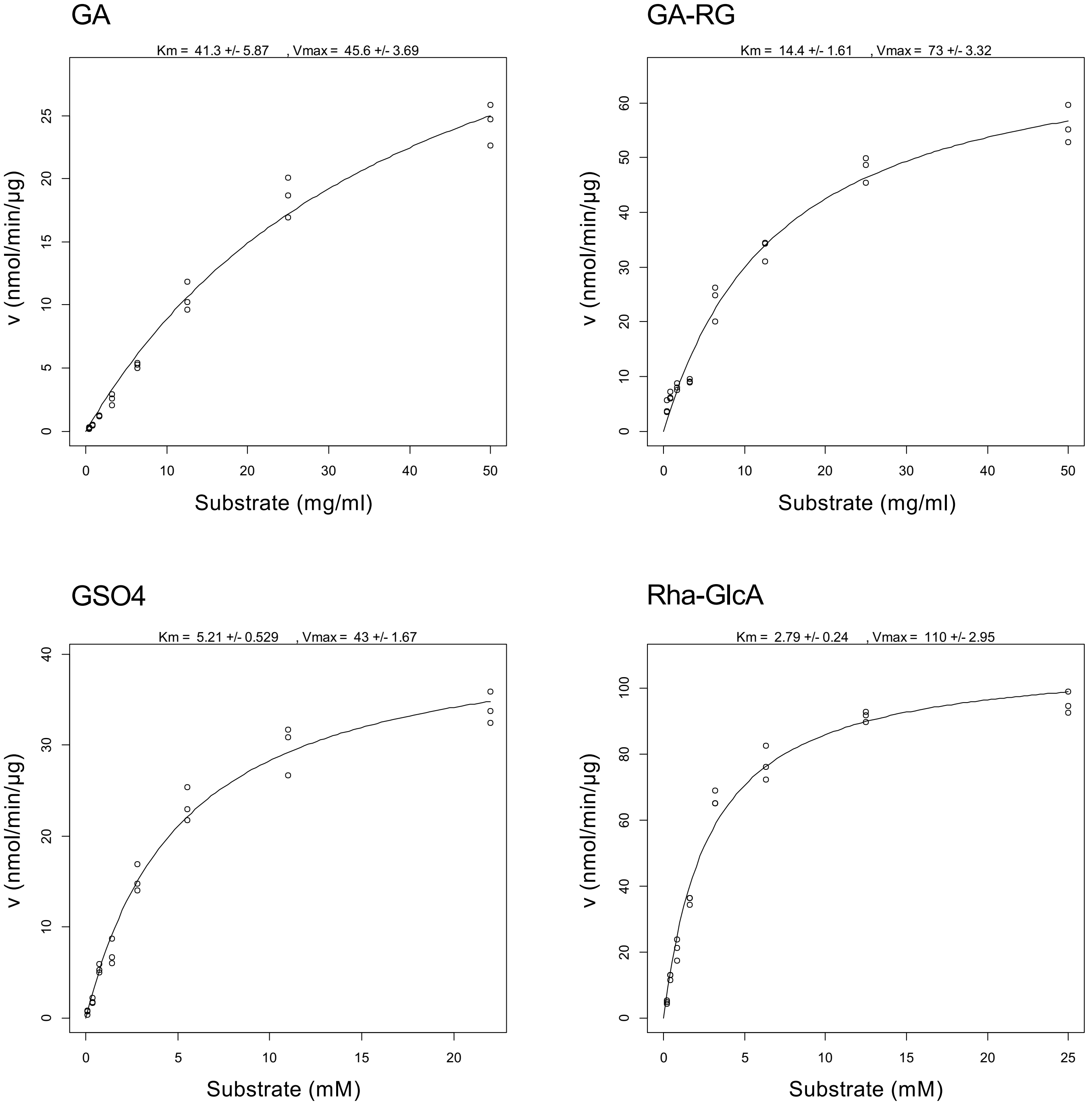

Figure S4. Kinetic analysis of FoRham1 using non-linear regression fitting. The kinetic parameters of FoRham1 were measured by the method described in the "Experimental procedures" section to determine the initial velocity for various concentrations of substrate. The data were fitted to a non-linear regression curve and the data were obtained from three independent experiments.

Supplementary Figure 5

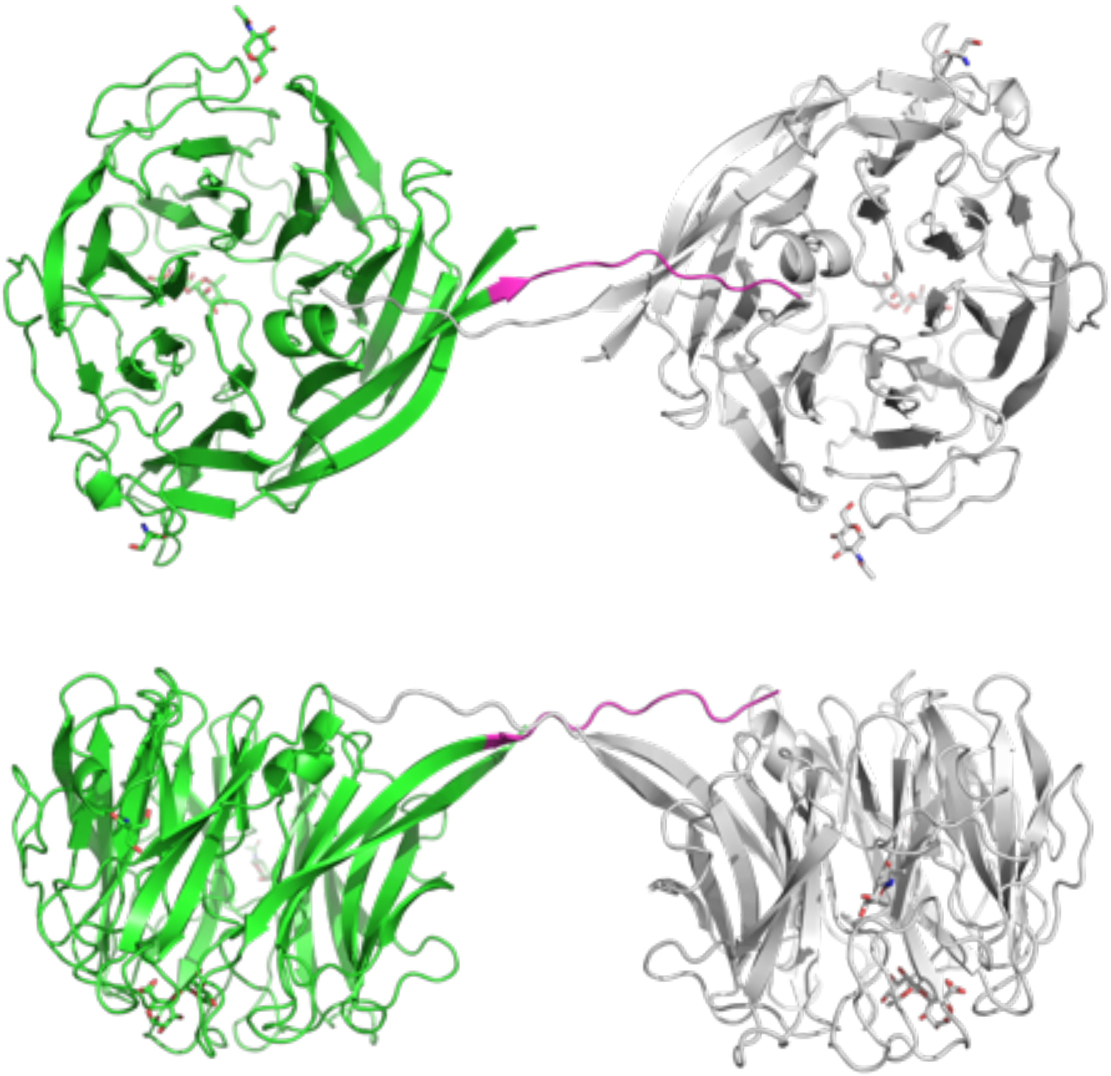

Figure S5. C-terminal swapping of H105F Rha-GlcA complex structure. The complex structure (green) and its crystallographic symmetry mate (gray) are shown as a ribbon model. The C-terminus of the complex structure (Glu421-Asn431) is shown in magenta.

Supplementary Figure 6

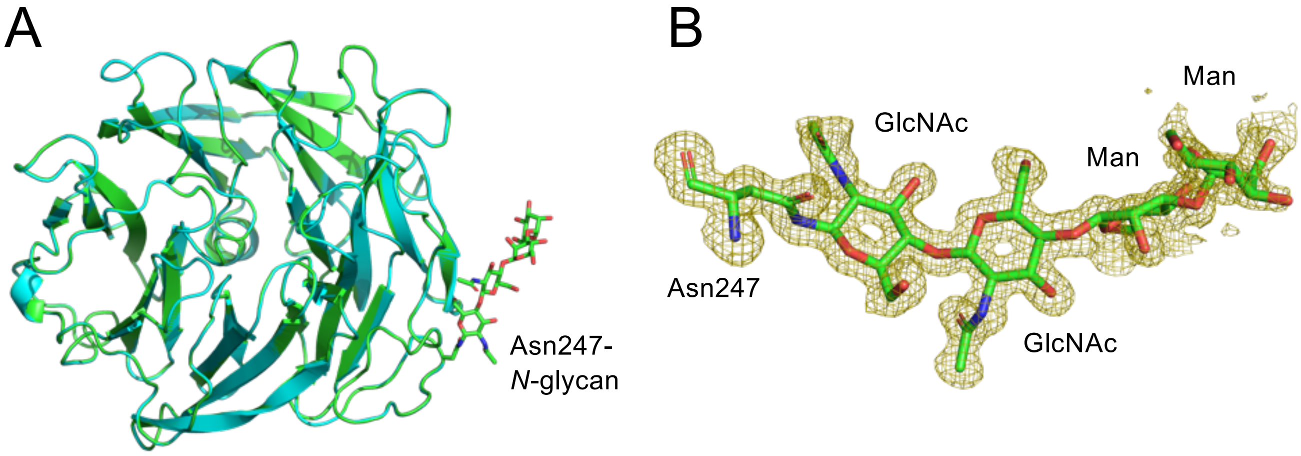

Figure S6. *N*-glycosylation. *A*, Superposition of WT (green) and N247A (cyan) mutant. *N*-glycans are shown as green sticks. *B*, The electron density of mF_o-F_c omit map (olive mesh, 3.0σ) of the *N*-glycan in FoRham1 WT.

Supplementary Figure 7

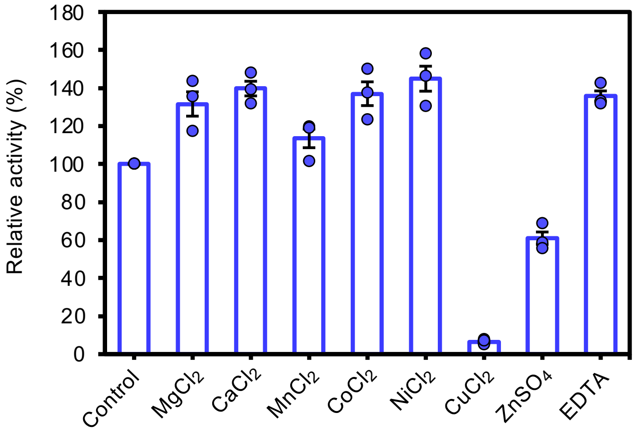

Figure S7. Effect of metal ions on the enzyme activity of FoRham1. Enzyme activity was measured using 0.1% GA as substrates in 50 mM HEPES-NaOH (pH 7.0) in the presence of 1 mM each compound. The reaction was performed at 30 °C for 5 min. After boiling the mixture for 3 min, released Rha were quantified by HPAEC-PAD. All experiments were performed in triplicate and were expressed as mean ± SE.

Supplementary Figure 8

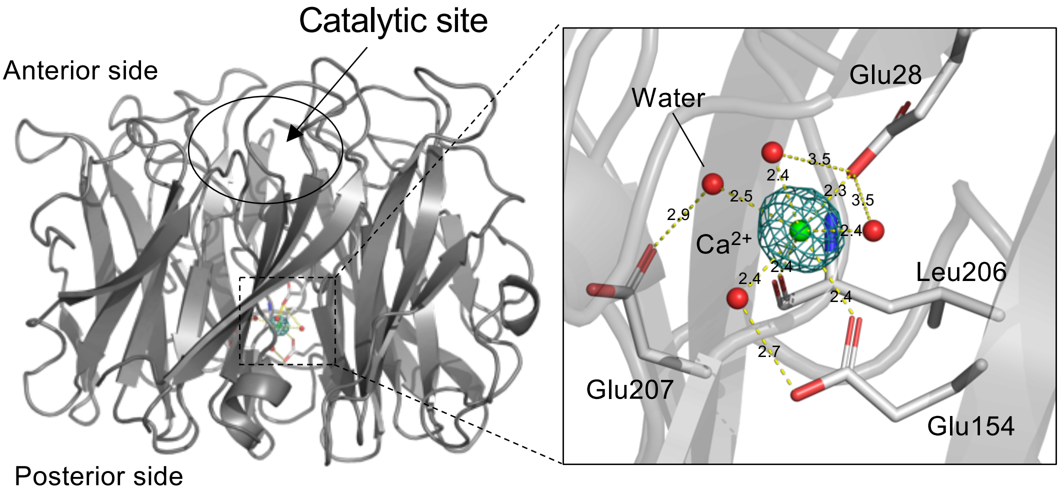

Figure S8. Ca^{2+} ion binding site of FoRham1 WT. Location of Ca^{2+} ion in the overall structure (left) and its mF_o-F_c omit electron density map (right, 3.0σ, blue mesh). Four metal-coordinating water molecules are shown as red spheres. Coordinating residues are shown as grey sticks, and the hydrogen bonds are shown as dotted yellow lines (< 3.5 Å).

Supplementary Figure 9

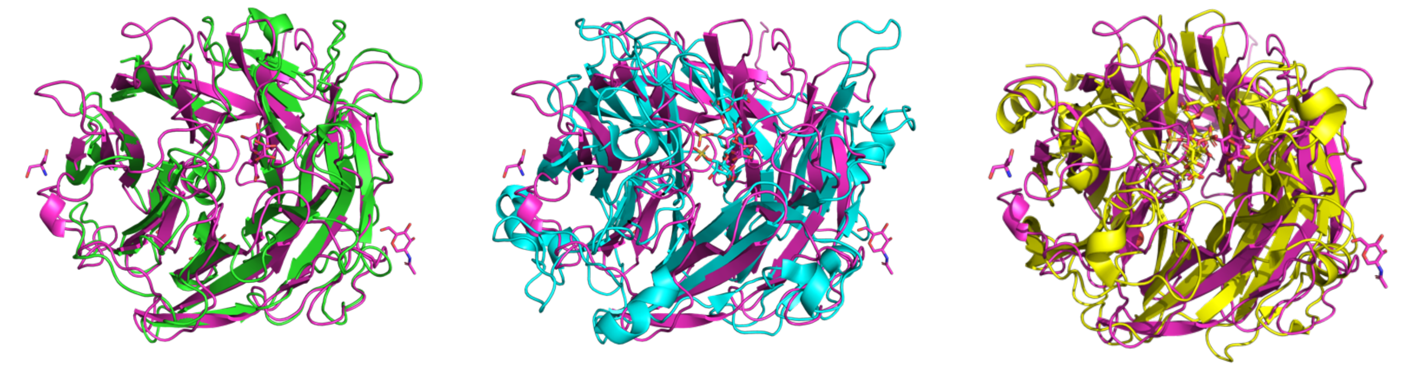

5MUL (GH145)
RMSD: 1.754Å for 252 Cα atoms

6BYT (PL24)
RMSD: 2.481Å for 301 Cα atoms

5UAS (PL25)
RMSD: 3.253 Å for 270 Cα atoms

Figure S9. Superimposition of FoRham1 with structural homologs. Structures of GH145 BT3686 with GlcA (left, green; PDB ID: 5MUL), PL24 LOR_107 with ulvan tetrasaccharide (middle, cyan; PDB ID: 6BYT), and PL25 PLSV_3936 with ulvan tetrasaccharide (right, yellow: PDB ID: 5UAS) are superimposed with FoRham1 Rha-GlcA complex (magenta). The bound ligands are shown as sticks. RMSD values with FoRham1 are as follows: 1.754 Å for 252 Cα atoms (5MUL), 2.481 Å for 301 Cα atoms (6BYT), and 3.253 Å for 270 Cα atoms (5UAS).

Table S1. Information on peptides identified based on MS/MS spectral data

Query	Peptide sequence	Precursor ion		Observed modification	Peptide score
		Charge	*m/z*		
140	MVNQEGQLIDTK	2	688.3466	None	31
142	IGQSGSGDSYIHR	3	459.5521	None	33
143	IGQSGSGDSYIHR	2	688.8278	None	47
144	IGQSGSGDSYIHR	3	459.7552	None	52
189	SLAEIPNTSTEPLFDK	2	881.4478	None	35

Table S2. Result of structural similarity search with the Dali server using the FoRham1 WT structure

PDB ID [a]	Z score	RMSD (Å)	LALI [b]	Identity (%)	Protein name	Organism	Activity	Family
5mum-A	47.6	2.3	377	29	BACINT_00347	*B. intestinalis*	None [c]	GH145 [d]
5mvh-A	47.3	2.1	374	28	BACCELL_00856	*B. cellulosilyticus*	α-L-Rhamnosidase	GH145 [d]
4irt-A	47.4	2.1	375	28	BACOVA_03493	*B. ovatus*	α-L-Rhamnosidase	GH145 [d]
5mul-A	43.2	2.1	356	27	BT3686	*B. thetaiotaomicron*	α-L-Rhamnosidase	GH145 [d]
5uas-B	33.3	2.9	362	16	PLSV_3936	*Pseudoalteromonas* sp. PLSV	Ulvan lyase	PL25
6byx-B	32.4	2.8	379	21	LOR_107	*Alteromonas* sp. LOR	Ulvan lyase	PL24
5c9p-A	26.9	3.0	308	12	PLL lectin	*P. luminescens*	L-Fucose binding	–

[a] Chain ID is shown after the hyphen.

[b] Number of aligned residues.

[c] Q48H mutant displayed α-L-rhamnosidase (hydrolase) activity toward gum arabic (12).

[d] GH145 will be renamed to PL42.

Table S3. The Mascot search parameters

Type of search	MS/MS ion search
Search engine	Mascot algorithm (2016/1/18)
Sequence database searched	NCBInr
Release date of sequence database searched	2016/1/14
Taxonomy	Fungi
Number of entries in the database searched	4,841,543
Protease used to generate peptides	Trypsin
Max missed cleavages	1
Fixed modification	Carbamidomethyl (C)
Variable modification	Oxidation (M)
Peptide mass tolerance	± 1.2 Da
Fragment mass tolerance	± 0.6 Da
Individual ions scores	> 56

Table S4. Sequences of primers for mutagenesis

Name	Sequence[a]
H85A (Fw)	5'-GATGGCGCTAACATGATCTCTATGGGT-3'
H85A (Rv)	5'-CATGTTAGCGCCATCCATCGTCTTCTG-3'
H85F (Fw)	5'-GATGGCTTTAACATGATCTCTATGGGT-3'
H85F (Rv)	5'-CATGTTAAAGCCATCCATCGTCTTCTG-3'
H105A (Fw)	5'-GATCATGCTGATGTCCCGATCAATTAT-3'
H105A (Rv)	5'-GCAATCAGCATGATCAAAGCTGAGGTG-3'
H105F (Fw)	5'-GATCATTTTGATGTCCCGATCAATTAT-3'
H105F (Rv)	5'-GCAATCAAAATGATCAAAGCTGAGGTG-3'
Y150A (Fw)	5'-CTGACGGCTCCGCGATTCGAGCCTCTG-3'
Y150A (Rv)	5'-TCGCGGAGCCGTCAGTGGCGAGTAAGG-3'
Y150F (Fw)	5'-CTGACGTTTCCGCGATTCGAGCCTCTG-3'
Y150F (Rv)	5'-TCGCGGAAACGTCAGTGGCGAGTAAGG-3'
R166A (Fw)	5'-GAGTTTGCTATTGGACAATCTGGGTCG-3'
R166A (Rv)	5'-TCCAATAGCAAACTCCAGTAGAAGGTC-3'
R166K (Fw)	5'-GAGTTTAAGATTGGACAATCTGGGTCG-3'
R166K (Rv)	5'-TCCAATCTTAAACTCCAGTAGAAGGTC-3'
S170A (Fw)	5'-GGACAAGCTGGGTCGGGCGATAGCTAC-3'
S170A (Rv)	5'-CGACCCAGCTTGTCCAATTCGAAACTC-3'
S170T (Fw)	5'-GGACAAACTGGGTCGGGCGATAGCTAC-3'
S170T (Rv)	5'-CGACCCAGTTTGTCCAATTCGAAACTC-3'
Y202A (Fw)	5'-CTGGATGCTTTGGACGGAAAGTTGTAT-3'
Y202A (Rv)	5'-GTCCAAAGCATCCAGTCCGTTGATGTA-3'
Y202F (Fw)	5'-CTGGATTTTTTGGACGGAAAGTTGTAT-3'
Y202F (Rv)	5'-GTCCAAAAAATCCAGTCCGTTGATGTA-3'
R220A (Fw)	5'-ACCGTCGCTGAAACACCGAACGCTGAT-3'
R220A (Rv)	5'-TGTTTCAGCGACGGTCCACGACGTATA-3'
R220K (Fw)	5'-ACCGTCAAGGAAACACCGAACGCTGAT-3'
R220K (Rv)	5'-TGTTTCCTTGACGGTCCACGACGTATA-3'
N247A (Fw)	5'-AATACGGCTGATACCAAGCTGACGAAA-3'
N247A (Rv)	5'-GGTATCAGCCGTATTGAACCATGTCTT-3'

[a] Underlines indicate mutation sites.
